# UPF2 Is a Critical Regulator of Liver Development, Function and Regeneration

**DOI:** 10.1371/journal.pone.0011650

**Published:** 2010-07-19

**Authors:** Lina A. Thoren, Gitte A. Nørgaard, Joachim Weischenfeldt, Johannes Waage, Janus S. Jakobsen, Inge Damgaard, Frida C. Bergström, Anna M. Blom, Rehannah Borup, Hanne Cathrine Bisgaard, Bo T. Porse

**Affiliations:** 1 Biotech Research and Innovation Centre (BRIC), University of Copenhagen, Copenhagen, Denmark; 2 Section for Gene Therapy Research, University of Copenhagen, Rigshospitalet, Copenhagen, Denmark; 3 Finsen Laboratory, University of Copenhagen, Rigshospitalet, Copenhagen, Denmark; 4 Department of Laboratory Medicine, Lund University, Malmö University Hospital, Malmö, Sweden; 5 Department of Clinical Biochemistry, University of Copenhagen, Rigshospitalet, Copenhagen, Denmark; 6 Department of Cellular and Molecular Medicine, Faculty of Health Sciences, University of Copenhagen, Copenhagen, Denmark; The Rockefeller University, United States of America

## Abstract

**Background:**

Nonsense-mediated mRNA decay (NMD) is a post-transcriptional RNA surveillance process that facilitates the recognition and destruction of mRNAs bearing premature terminations codons (PTCs). Such PTC-containing (PTC+) mRNAs may arise from different processes, including erroneous processing and expression of pseudogenes, but also from more regulated events such as alternative splicing coupled NMD (AS-NMD). Thus, the NMD pathway serves both as a silencer of genomic noise and a regulator of gene expression. Given the early embryonic lethality in NMD deficient mice, uncovering the full regulatory potential of the NMD pathway in mammals will require the functional assessment of NMD in different tissues.

**Methodology/Principal Findings:**

Here we use mouse genetics to address the role of UPF2, a core NMD component, in the development, function and regeneration of the liver. We find that loss of NMD during fetal liver development is incompatible with postnatal life due to failure of terminal differentiation. Moreover, deletion of *Upf2* in the adult liver results in hepatosteatosis and disruption of liver homeostasis. Finally, NMD was found to be absolutely required for liver regeneration.

**Conclusion/Significance:**

Collectively, our data demonstrate the critical role of the NMD pathway in liver development, function and regeneration and highlights the importance of NMD for mammalian biology.

## Introduction

The NMD machinery is part of a larger set of RNA surveillance pathways that check the integrity of mRNAs before rendering them available for translation in the cytoplasm. Specifically, NMD recognizes mRNAs containing PTCs and routes them towards degradation, thereby providing molecular means to avoid the production of C-terminally truncated protein from damaged mRNAs [1], [2].

The molecular rules by which stop codons are recognized as premature have been elucidated in organisms ranging from yeast to man. In mammals, the exon junction complex (EJC), which is a protein complex deposited upstream of the exon-exon junction during splicing, has long been considered as the marker distinguishing premature from normal stop codons [2], [3]. As most mammalian genes carry their termination codon in the last exon, a stop codon located upstream of an EJC marks it as premature. Whereas this model explains many features of mammalian NMD, it fails to explain why some stop codons without a downstream EJC are recognized as PTCs. Recently, it has been demonstrated that also the distance of the stop codon to the poly(A) tail can determine whether a stop codon is recognized as normal or premature [4], [5], [6].

PTC-containing (PTC+) “erroneous” mRNAs may arise from transcription of pseudogenes, from nucleotide misincorporation during transcription of *bona fide* genes, or more frequently due to processing errors during the posttranscriptional maturation of the primary transcript [7], [8]. In addition to serve as a “filter” of genetic noise, the NMD machinery has also been described as an important regulator of mRNA output. Most prominent is its role in a process termed AS-NMD, in which an alternative splicing event results in the generation of two mRNA isoforms, one of them being NMD sensitive [9], [10], [11]. Intriguingly, AS-NMD is particular frequent among splicing factors and the ability of some of these proteins to regulate alternative splicing events within their own mRNAs sets up a series of autoregulatory loops by which homeostasis of these factors are maintained [9], [10]. Obviously, disruption of these loops, for instance by ablation of NMD, may have profound effects on alternative splicing in general. Finally, the NMD pathway has also been reported to modulate the expression of several normal genes by mechanisms involving the presence of an upstream open reading frame or an intron in the 3′ untranslated region, both of which are predicted to elicit NMD [12], [13]. To what extent this is used in a regulatory context is not clear, but it has been proposed that different tissues display different efficiencies of NMD, which in turn may lead to tissue-specific differences in mRNAs containing NMD features [14].

The molecular components of the NMD machinery in mammals have been reported to consist of at least 9 members: UPF1, UPF2 and UPF3a/b constitutes the core NMD complex and is conserved from yeast to man [2]. SMG1, SMG5 and SMG7 are later evolutionary additions to the NMD machinery and are involved in a phosphorylation/dephosphorylation cycle of UPF1 necessary for NMD [15]. SMG6 has recently been described as an endonuclease involved in cleavage of NMD substrates, whereas the functions of the newest members of NMD machinery SMG8 and SMG9 are only partially defined [16], [17]. Besides their established importance in the NMD process *per se*, NMD components have also been reported to play roles in other cellular pathways. These include the DNA damage response (SMG1, UPF1 [15], [18]), cell cycle progression (SMG1, UPF1, SMG6 [18], [19]) and in telomere maintenance (SMG1, UPF1, SMG6 [20]).

Whereas the NMD pathway has been extensively studied in tissue-culture we have very limited insights into its roles in the context of intact mammalian organisms. Mice lacking either UPF1 or UPF2 die early during embryonic development and have therefore not been amenable to detailed analysis [13], [21]. Recently, we reported on the characterization of mouse lines lacking UPF2 in various hematopoietic compartments [13]. Here we uncovered an essential role for UPF2 in proliferating cells and in degrading PTC+ transcripts from genomic rearrangement by-products during lymphoid development. Furthermore, we showed that the NMD machinery regulates the expression of specific subsets of genes including snoRNA host genes and is instrumental in AS-NMD *in vivo*. Despite these findings we completely lack knowledge on the role of NMD in other tissues.

The mammalian liver is a complex organ, which plays several different roles during embryonic development and in adult life. During fetal life the developing liver serves as the main hematopoietic organ, whereas its primary function in the adult is metabolic processes, including detoxification, glycogen storage as well as hormone and plasma protein production.

Liver development in the mouse initiates at embryonic day (E) 8.5 from an area of the primitive gut endoderm that is specified by signals from the cardiac mesoderm and the surrounding mesenchyme [22]. These signals ultimately result in the proliferation of hepatoblasts followed by their migration into the surrounding mesenchyme. At the later stages of liver development, hepatoblasts terminally differentiate into hepatocytes and cholangiocytes, the two hepatic cell lineages, in a process involving epithelial transformation orchestrated by hepatocyte nuclear factor 4α (HNF4α) [23].

The adult mouse liver has served as an experimental paradigm for tissue regeneration for decades. Surgical removal of 70% of the liver mass, in a process termed partial hepatechtomy (PH), leads to a coordinated cell cycle entry of hepatocytes in the remaining lobes resulting in the compensatory outgrowth of a full-sized functional liver. Genetic dissection in mice has identified the importance of several members of the innate immune system in the so-called priming phase of PH, including IL-6 and members of the complement system. Disruption of IL-6, C3 and C5 significantly compromise or delay liver regeneration mainly by blunting activation of the STAT3 pathway [24], [25]. Signalling through STAT3 is only transient and is in part down-regulated by SOCS 3 (a target of STAT3) in a negative feed-back loop, hypothesized to protect cells from prolonged cytokine signaling [26]. The priming phase of PH ultimately drives hepatocytes into active cell cycle with DNA synthesis peaking at 36–44 hours.

Here we set out to test the importance of the NMD component UPF2 in a tissue of endodermal origin. Specifically, we use our conditional UPF2 *null* mouse line to probe the functional role of NMD at several stages of the “life cycle” of the liver, including fetal development, adult life and in a regenerative setting. We find that loss of UPF2 leads to activation of the DNA damage response pathway in the developing liver and that UPF2 is essential for development of a liver capable of sustaining postnatal life. Disruption of UPF2 in the adult liver interferes with tissue homeostasis and result in extensive liver damage. Finally, UPF2 *null* hepatocytes fail to enter active cell cycling following PH again eluding to an important role for UPF2 in proliferating cells.

## Results

### Ablation of NMD in the developing liver leads to perinatal lethality

Mice deficient of the core NMD component UPF2 die *in utero* around E3.5-E7.5 [13]. In order to circumvent this early embryonic lethality and to be able to probe the function of NMD during the development of a defined organ, we crossed the conditional *Upf2^fl/+^* allele onto the *Alfp-Cre* strain thereby facilitating deletion of UPF2 during liver development. In the *Alfp-Cre* line, expression of Cre recombinase is driven by regulatory elements from both the albumin promoter and the Alfa-fetoprotein enhancer, thereby leading to Cre expression in the developing liver endoderm from around E10.0 [27].

**Figure 1:**
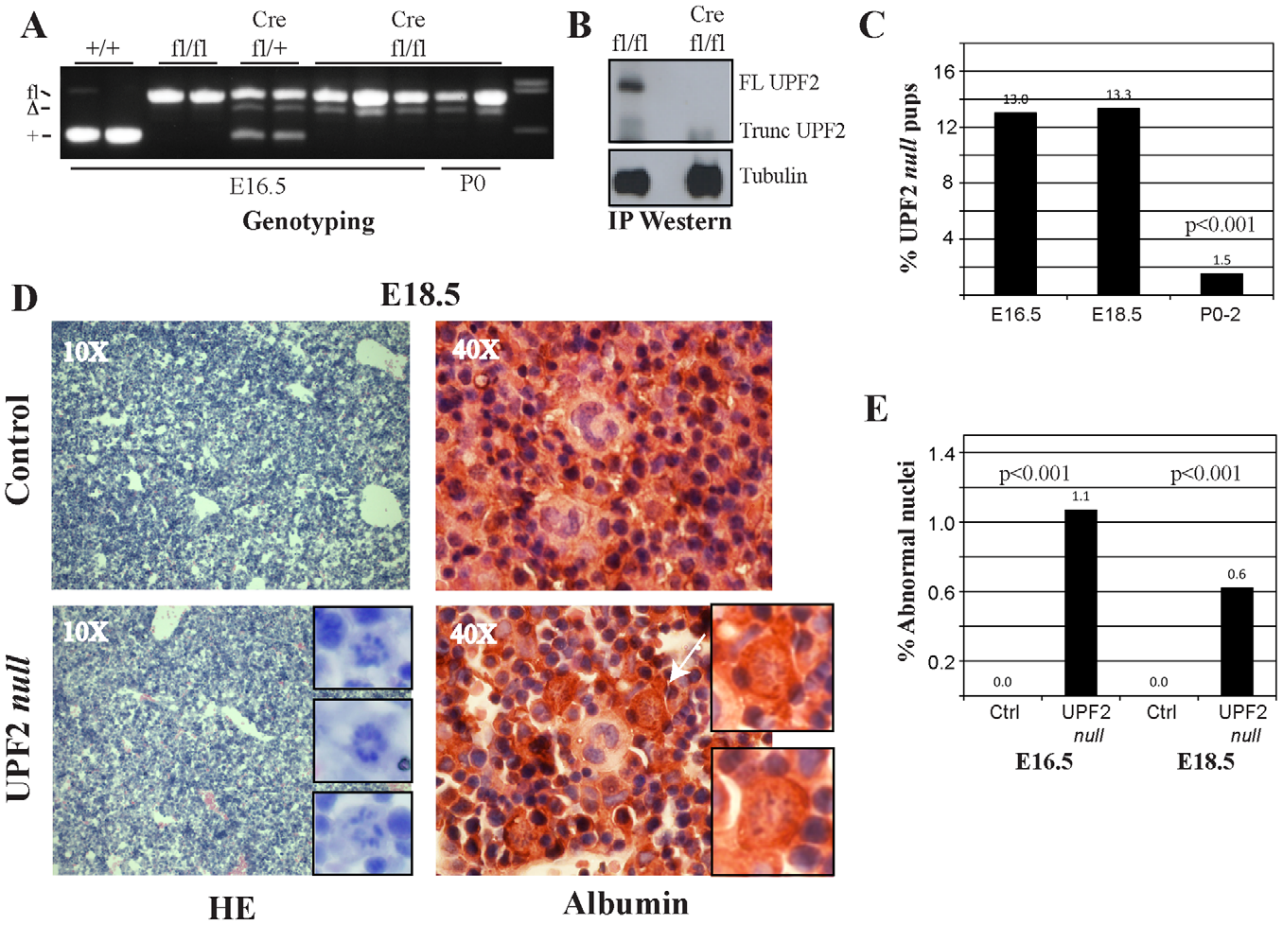
Loss of UPF2 during fetal liver development. (A) Genotyping of embryos and newborns from intercrosses between *Upf2^fl/+^; Alfp-Cre* males and *Upf2^fl/+^* females. (B) Western blot analysis of UPF2 and α-tubulin immunoprecipitates from *Upf2^fl/fl^* and *Upf2^fl/fl^; Alfp-Cre* E16.5 embryos. (C) Percentages of UPF2 *null* (defined as *Upf2^fl/fl^; Alfp-Cre*) pups found at E16.5 (n = 146), E18.5 (n = 90) and P0-2 (n = 137). (D) Histological analysis of control (*Upf2^fl/f^*) and UPF2 *null* (*Upf2^fl/fl^; Alfp-Cre*) E18.5 fetal livers stained with HE (inset showing nuclei with abnormal morphology) or with an anti-Albumin antibody (white arrow indicates a nucleus with abnormal morphology, see insert). (E) Quantification of nuclei with abnormal morphology at E16.5 and E18.5 of control (*Upf2^fl/f^*) and UPF2 *null* (*Upf2^fl/fl^; Alfp-Cre*) fetal livers. The data represent quantification of at least 2000 cells from 3 different individuals of each genotype at each of the two time points (mean +/− standard deviation).

Intercrosses between *Upf2^fl/+^; Alfp-Cre* males and *Upf2^fl/+^* females yielded *Upf2^fl/fl^; Alfp-Cre* fetuses in the expected frequencies (1 in 8) at E13.5 (not shown), E16.5 and E18.5 (Figure 1C). In contrast we only recovered 1.5% (2/137) *Upf2^fl/fl^; Alfp-Cre* P0-P2 newborns demonstrating that liver-specific loss of UPF2, and by inference NMD, leads to perinatal lethality (Figure 1C). When genomic DNA from *Upf2^fl/fl^; Alfp-Cre* fetuses was analyzed for deletion of the floxed exons 2–3 of *Upf2*, we observed only partial deletion (Figure 1A) accompanied with loss of full-length UPF2 protein and the appearance of a truncated non-functional isoform, as reported earlier (Figure 1B) [13]. The partial recombination that we observe in *Upf2^fl/fl^; Alfp-Cre* embryos is due to the major contribution of hematopoietic cells to the fetal liver mass at these stages of development.

Loss of UPF2 was not accompanied by macroscopic changes in liver attributes suggesting that UPF2 is not essential for the liver to reach its final size (data not shown). Consistently, histological analysis of E16.5 (Figure S1) and E18.5 (Figure 1D) did not reveal any gross changes in tissue morphology in the *Upf2^fl/fl^; Alfp-Cre* fetal livers, however we did observe nuclei with abnormal morphology at both these developmental stages. These abnormal nuclei were present in frequencies of 0.6% and 1.1% of E18.5 and E16.5 *Upf2^fl/fl^; Alfp-Cre* fetal livers, respectively, and were not found in sections from control littermates (Figure 1E). Moreover, the hepatocyte origin of the cells with abnormal nuclei was demonstrated by staining with an albumin specific antibody (Figure 1D).

### UPF2 deficient cells with abnormal nuclei are arrested in mitosis

**Figure 2:**
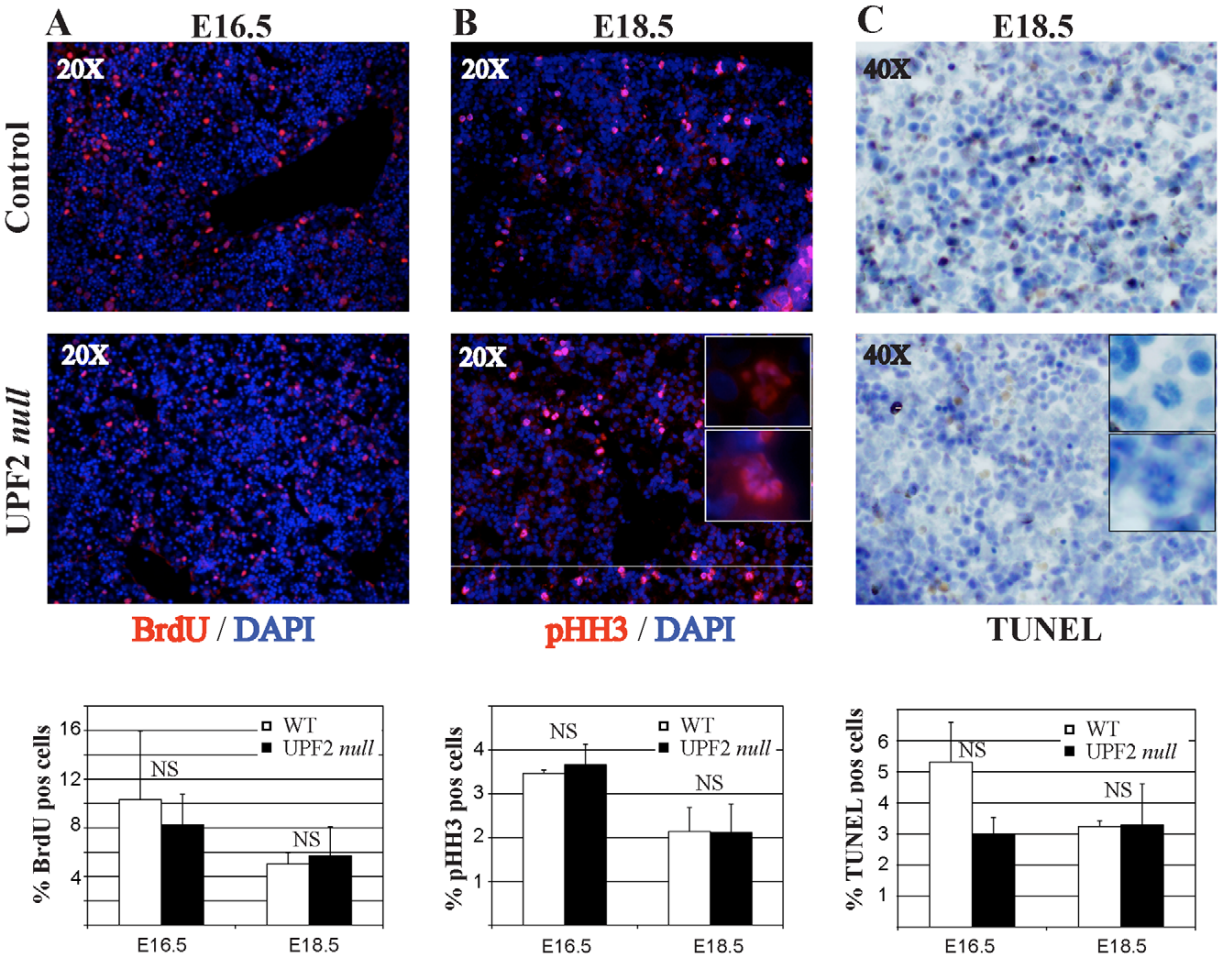
UPF2 loss during liver development does not affect proliferation, number of mitotic cells or apoptosis. (A) UPF2 *null* fetal livers incorporate BrdU to similar extent as control livers. (B) UPF2 *null* fetal livers have similar number of mitotic cells as assayed by phosphorylation of S10 of histone H3. Insert shows that nuclei with abnormal morphology are positive for pHH3. (C) TUNEL staining of UPF2 *null* and control fetal livers. Inserts shows that nuclei with abnormal morphology are TUNEL-negative. The data represent quantification of at least 2000 cells from 3 different individuals of each genotype at each of the two time points (mean +/− standard deviation).

In order to further characterize the phenotype induced by liver-specific loss of UPF2 during embryonic development, and in particular the cells with abnormal nuclear morphology, we next addressed the proliferative as well as the apoptotic status of fetal liver cells of E16.5 and E18.5 embryos (Figure 2, Figure S1). Consistent with the absence of loss of liver mass, both control and UPF2 deficient embryos incorporated the S-Phase specific marker BrdU to the same extent (Figure 2A). Similarly, the numbers of pHH3 positive mitotic cells were identical for both control and UPF2 deficient embryos (Figure 2B). However, a significant fraction of pHH3 positive cells in UPF2 deficient fetal livers displayed the abnormal nuclear morphology at both E16.5 (12%+/−3%, data not shown) and E18.5 (13%+/−4%, Figure 2B, insert). Furthermore, all abnormal nuclei in UPF2 deficient fetal livers stained positive for the mitotic marker pHH3, strongly suggesting that these cells are either arrested or at least retarded in mitosis. Finally, we tested the possibility that the UPF2 deficient abnormal nuclear cells were lost by apoptosis. However, TUNEL (Terminal deoxynucleotidyl transferase dUTP nick end labeling) staining failed to reveal evidence for apoptosis neither in these cells, nor in the overall levels of apoptotic cells in UPF2 deficient livers (Figure 2C, Figure S1).

Collectively these findings demonstrate that loss of UPF2 during fetal liver development leads to the appearance of cells arrested in mitosis displaying abnormal nuclear morphology. However, the loss UPF2 has no impact on overall proliferation or cellular turnover.

### Loss of UPF2 impairs the full development of the fetal liver and leads to activation of the DNA Damage pathway

**Figure 3:**
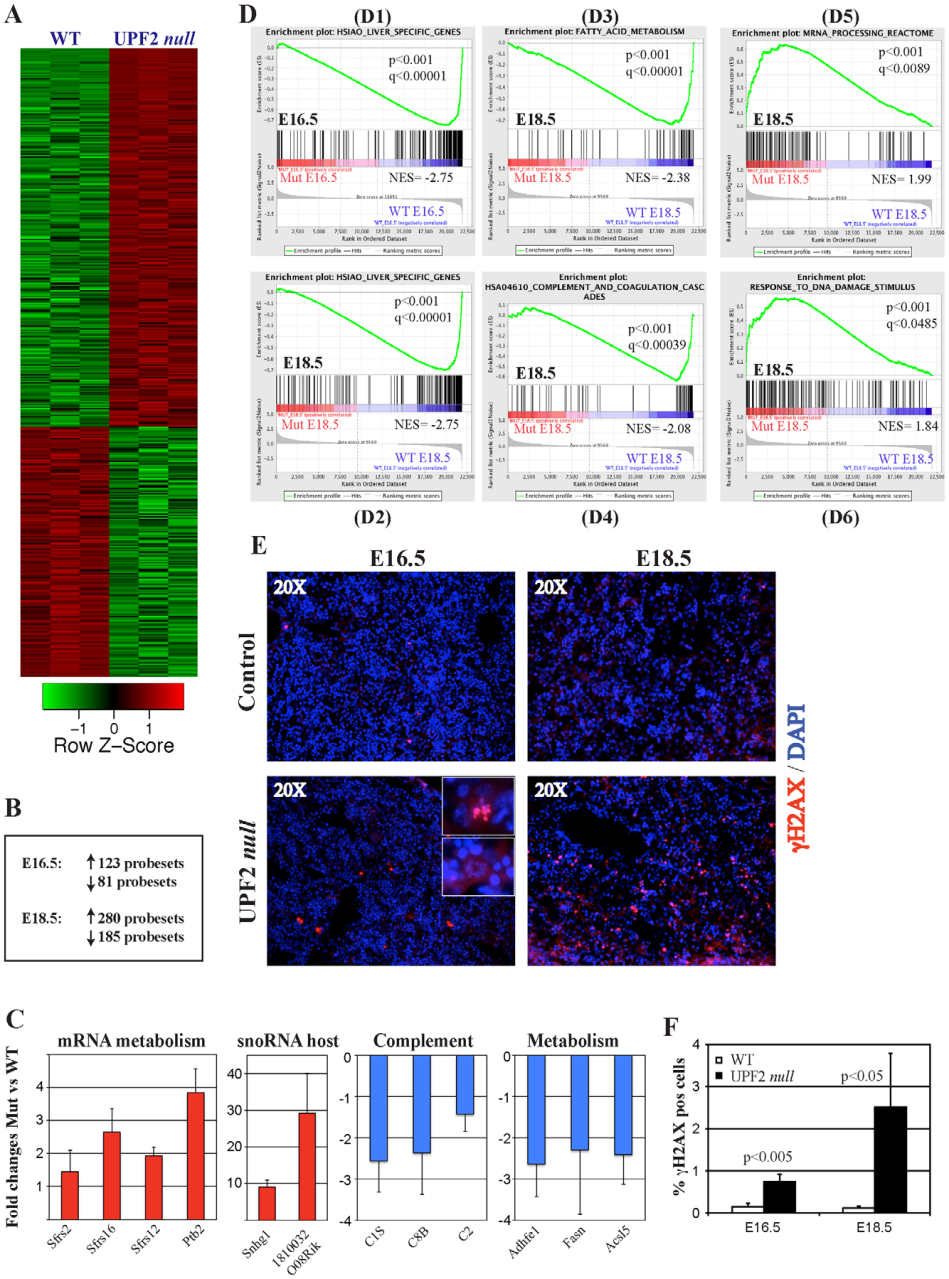
Gene expression analysis of control and UPF2 null fetal livers. (A) Cluster analysis of normalized affymetrix expression data from control and UPF2 *null* E18.5 fetal livers. (B) Summaries of deregulated probesets at E16.5 and E18.5 in control *vs* UPF2 *null* fetal livers. (C) qRT-PCR validation of deregulated genes belonging to different functional classes in E18.5 UPF2 *null* fetal livers. Red and blue colors indicate up-regulated and down-regulated genes, respectively, in UPF2 *null* fetal livers. cDNA was synthesized from 3 mice of each genotype and assayed by qPCR in triplicates (mean +/− standard deviation). (D) Selected enriched gene sets from GSEA analysis. D1-D2: HSIAO_Liver_Specific_Genes, D3: Fatty_Acid Metabolism, D4: HSA04610_Complement_And_Coagulation_Cascades, D5: mRNA_Processing_Reactome, D6: Response_to_DNA_Damage_Stimulus. Normalized enrichment scores, p-value and q-values (FDR) are indicated for each gene set. Negative and positive NES values indicate enrichment in control and UPF2 *null* fetal livers, respectively. Y-axes indicate Enrichment scores (top) and Ranked list metric (bottom). Green lines indicate enrichment profile, black bars are hits (i.e. genes) and grey profiles are ranked metric score. Red and blue indicate positive and negative correlations, respectively, with the indicated gene sets. (E) Immunofluorescence analysis of control and UPF2 *null* fetal livers demonstrate the accumulation of nuclei positive for the DNA Damage marker γH2AX upon loss of UPF2. Insert shows a close-up on two nuclei with abnormal morphology, one of them being positive for γH2AX. (F) Quantification of the data in (E). The data represent quantification of at least 2000 cells from 3 different individuals of each genotype at each of the two time points (mean +/− standard deviation).

The appearance of nuclei with abnormal morphology does not fully explain the perinatal lethality induced by liver-specific ablation of UPF2. In order to address the cause of the perinatal lethality in more detail, we subjected RNA from E16.5 and E18.5 fetal livers to global gene expression profiling. Comparison of the resultant expression profiles identified a total of 204 and 465 probesets at E16.5 and E18.5, respectively, which were deregulated (fold changes >2 or <−2; P<0.05) upon loss of UPF2 in fetal liver cells (Figure 3A,B; Table S1 for full lists of deregulated genes). Previously we have shown that loss of UPF2 in hematopoietic cells leads to up-regulated expression of snoRNA host genes, which is a class of transcripts that encodes snoRNA mainly in their intervening introns [13]. Similarly to the case in hematopoietic cells, a major fraction of the genes up-regulated in UPF2 deficient livers at E16.5 and E18.5 were snoRNA host genes (13 and 15 genes, respectively; See Tables S1,2), suggesting that NMD is a major regulator of this pathway in several different cell types.

As mice lacking HNF4α in the developing liver fail to undergo terminal differentiation due to defects in epithelial transformation we compared the expression of selected genes in our fetal UPF2 *null* livers to those reported in HNF4α *null* cells [23]. Consistent with the normal morphology of the E18.5 UPF2 *null* livers, *Hnf4a* and its downstream epithelial target genes *Cdh1, Ceacam1, Gjb1 and Gjb2* were either unchanged or marginally down-regulated in UPF2 *null* livers (Table S3) These findings demonstrate that loss of UPF2 interfere with liver development at a stage beyond the HNF4α-dependent epithelial transformation.

We next subjected the E16.5 and E18.5 datasets to Gene Set Enrichment Analysis (GSEA) with the aim to identify pathways that were selectively deregulated by loss of UPF2 in fetal liver [28]. Intriguingly, gene sets representing “Liver-specific Genes” correlated negatively with UPF2 *null* fetal livers demonstrating that loss of UPF2 has a global effect on the development of the liver (Figure 3D). Specifically a range of metabolic pathways, including “Fatty Acid Metabolism”, “Valine Leucine and Isoleucine metabolism”, “Steroid Biosynthetic Process” and more were down-regulated in UPF2 *null* fetal livers both at E16.5 and E18.5 (Figure 3C,D and Table S4 for the full analysis). In addition, we noticed a decrease in the expression of genes belonging to “HSA04610 Complement and coagulation cascades” suggesting that absence of NMD in the developing liver might influence the function of this system (Figure 3C,D). GSEA also revealed pathways in which the loss of NMD in fetal liver cells resulted in an up-regulated expression of genes involved in mRNA processing (“mRNA processing reactome”), including splicing factors (Figure 3C,D) which have previously been shown to be auto-regulated via NMD [9], [10], [13], [29]. These data are consistent with a role of NMD in regulating splicing homeostasis.

Finally, GSEA also indicated that UPF2 deficient fetal livers up-regulated transcripts induced by DNA damage (“Response to DNA damage stimulus” Figure 3C,D). An independent marker for DNA damage is γH2AX, which is phosphorylated upon DNA damage and accumulates at double-stranded DNA breaks. Indeed, γH2AX-positive cells accumulated in UPF2 deficient livers at both E16.5 and E18.5 demonstrating that loss of UPF2 activates the DNA damage response pathway (Figure 3E,F).

In summary, our gene expression analysis indicates that ablation of UPF2 during fetal organogenesis is incompatible with the development of a metabolically functional competent liver able to sustain postnatal life.

### Ablation of NMD in adult quiescent liver disrupts liver homeostasis

**Figure 4:**
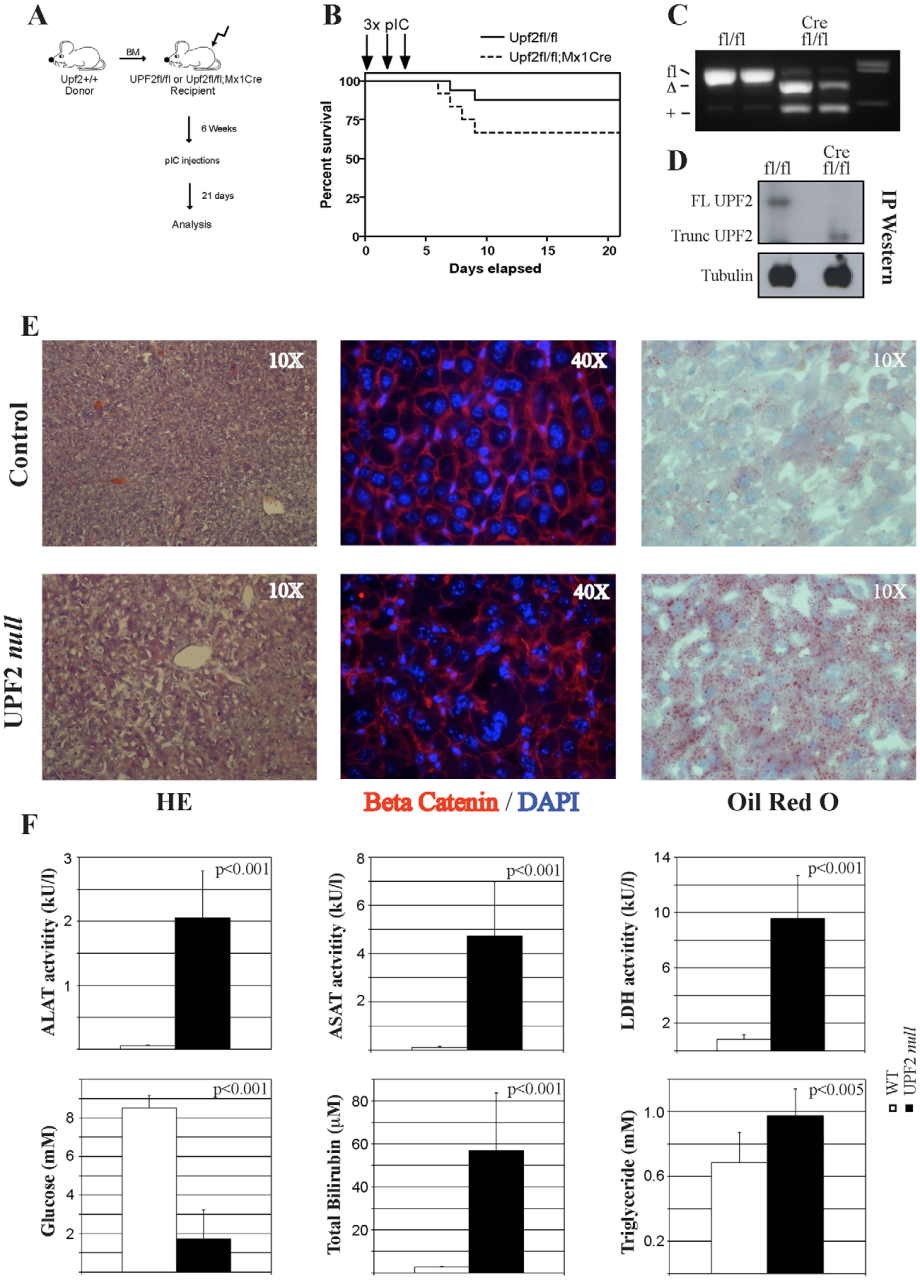
UPF2 is essential for homeostasis of the adult liver. (A) Experimental strategy for the induction of UPF2 loss in the adult liver of BM transplanted control (*Upf2^fl/fl^*) and *Upf2^fl/fl^;Mx1Cre* mice. (B) BM transplantation rescues the lethality of *Upf2^fl/fl^;Mx1Cre* mice. (C) Genotyping of BM transplanted (control and UPF2 *null* mice 3 weeks after the first pIC injection demonstrates efficient deletion in liver. UPF2 *null* adult livers also accumulate WT (+) cells of hematopoietic origin. (D) Western blot analysis of UPF2 and α-tubulin immunoprecipitates from control and UPF2 *null* adult livers. (E) Histological analysis using either immunohistochemistry of immunofluorescence techniques demonstrated loss in tissue organization (HE and β-catenin) and accumulation of lipids (Oil Red O) upon loss of UPF2 in adult liver. (F) Serum analysis of control and UPF2 *null* adult livers shows the accumulation of markers for liver damage (ALAT, ASAT, LDH and bilirubin), lowered glucose levels and triglyceride accumulation. The data represent the average of >8 mice in each group (mean +/− standard deviation).

We next wanted to test if the NMD pathway is uniquely important during fetal liver development or whether it also plays a role during adulthood in a steady-state quiescent liver. To that end we used the *Mx1-Cre* transgenic line in which Cre expression can be induced by injection of mice with the double-stranded RNA polyinosinic-polycytidylic acid (pIC), resulting in Cre-mediated recombination mainly in liver and in hematopoietic cells [30]. In order to circumvent the strong lethality induced by loss of UPF2 in the hematopoietic compartment, we adopted an experimental strategy where we generated chimeric mice by transplanting WT bone marrow (BM) cells into lethally irradiated *Upf2^fl/fl^* control and *Upf2^fl/fl^; Mx1Cre* experimental animals [13] and, following reconstitution of the hematopoietic system, induced loss of UPF2 in liver cells by injection with pIC (Figure 4A). Although this strategy efficiently rescues mice from the early hematopoietic associated lethality (median survival time  = 6 days, [13]), they did become moribund after 3 weeks suggesting a crucial role of UPF2 in the adult liver (Figure 4B and data not shown). Analysis of UPF2 *null* livers 21 days post pIC revealed efficient excision at the *Upf2* locus resulting in the expression of the truncated non-functional form of UPF2, as reported previously (Figure 4C,D; [13]). This was accompanied by strong disorganization of the liver tissue and by the induction of hepatic steatosis as evidenced by accumulation of lipids in *Upf2 null* livers (Figure 4E). Further evidence supporting the notion that loss of UPF2 leads to liver damage comes from analysis of serum from UPF2 *null* mice 21 days post pIC injection. Here, loss of UPF2 resulted in highly elevated serum levels of markers of liver damage including Alanine Aminotransferase (ALAT), Aspartate Aminotransferase (ASAT) and Lactate Dehydrogenase (LDH) activites, accompanied by low glucose levels, increased triglyceride values and highly elevated levels of bilirubin (Figure 4F). Surprisingly, but consistent with the data from the fetal liver system, loss of UPF2 did not lead to any appreciable cell death as assayed by TUNEL staining (Figure S2). Moreover, and in contrast to the effects in fetal liver, we were unable to detect any induction of DNA damage upon deletion of UPF2 in the adult liver (data not shown). These data demonstrate that UPF2, and by inference the NMD system, is essential for maintenance of liver homeostasis and that liver-specific loss of UPF2 leads to pronounced liver damage and death within a month after its deletion.

**Figure 5:**
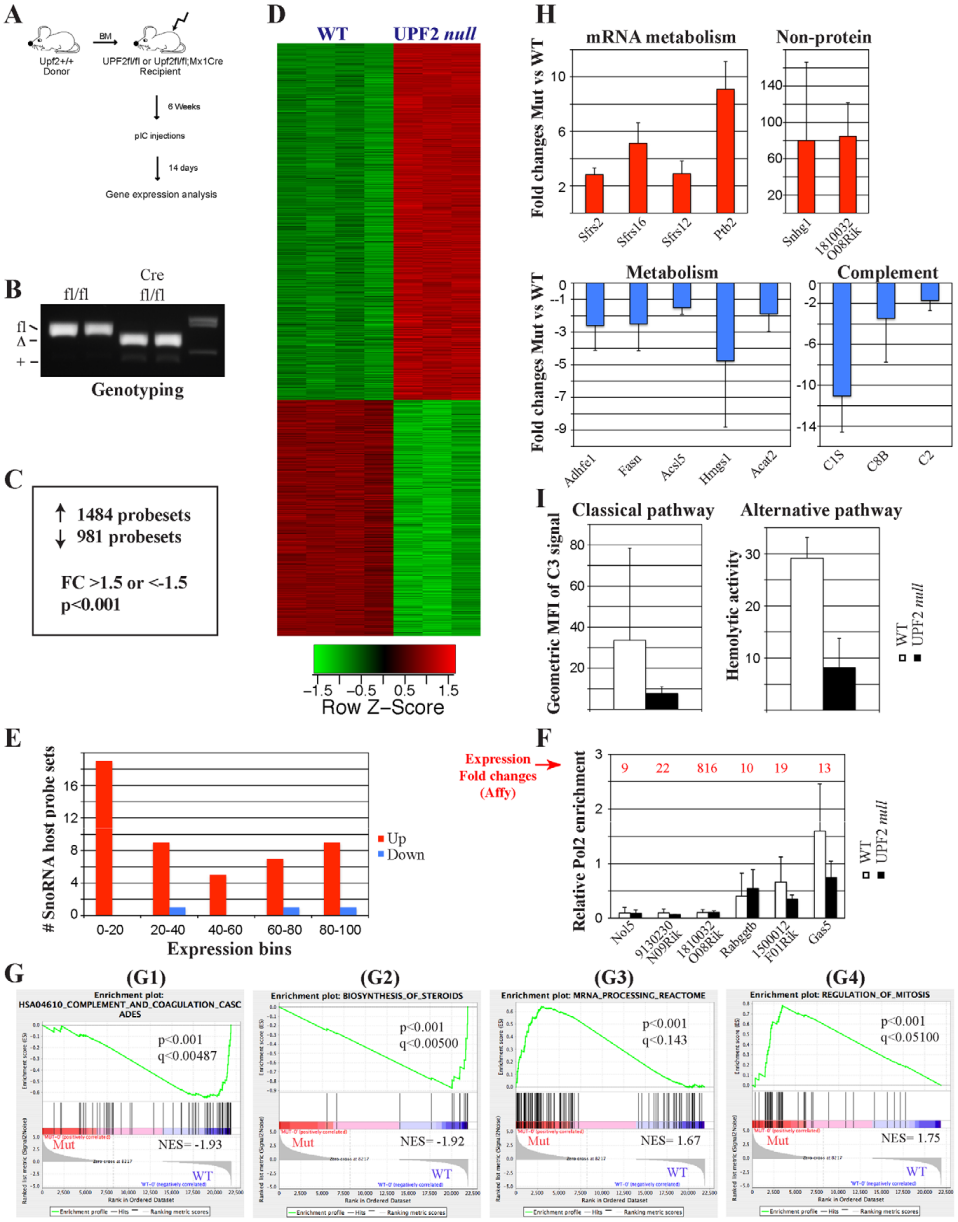
Gene expression analysis of UPF2-deficient adult livers. (A) Experimental strategy for the induction of UPF2 loss in the adult liver of BM transplanted control (*Upf2^fl/fl^*) and *Upf2^fl/fl^; Mx1Cre* mice. (B) Genotyping of control and UPF2 *null* mice 2 weeks after the first pIC injection demonstrates efficient deletion in liver. (C) Summaries of deregulated probesets in control and UPF2 *null* adult livers. (D) Cluster analysis of normalized affymetrix expression data from control and UPF2 *null* adult livers. (E) Distribution of snoRNA host genes (defined as genes having intron-encoded snoRNAs) among up and down-regulated genes. 0–20, 20–40 etc. indicates percentiles within up and down-regulated probesets. Thus, 0–20 indicates the top 20% of up and down-regulated genes. (F) ChIP analysis of RNA Pol2 occupancy of selected snoRNA host genes. Enrichment values are given relative to *Actb* (normalized to a gene dessert). Expression fold changes (all up in UPF2 *null* livers) derived from affymetrix gene expression profiling are shown in red above each gene. Immunoprecipitates were performed on two mice of each genotype and assayed by qPCR in triplicates (mean +/− standard deviation). (G) Selected enriched gene sets from GSEA analysis. G1: HSA04610_Complement_And_Coagulation_Cascades, G2: Biosynthesis_of_steroids, G3: mRNA_Processing_Reactome, G4: Regulation_of_mitosis. Normalized enrichment scores, p-value and q-values (FDR) are indicated for each gene set. Negative and positive NES values indicate enrichment in control and UPF2 *null* adult livers, respectively. Y-axes indicate Enrichment scores (top) and Ranked list metric (bottom). Green lines indicate enrichment profile, black bars are hits (i.e. genes) and grey profiles are ranked metric score. Red and blue indicate positive and negative correlations, respectively, with the indicated gene sets. (H) qRT-PCR validation of deregulated genes belonging to different functional classes in adult UPF2 *null* livers. “Non-protein” indicates SnoRNA host genes. Red and blue colors indicate up-regulated and down-regulated genes, respectively, in UPF2 *null* adult livers. cDNA was synthesized from 3 mice of each genotype and assayed by qPCR in triplicates (mean +/− standard deviation). (I) Functional assessment of the classical and alternative complement pathways in control and UPF2 *null* adult sera. The data represent the average of 3 experiments each involving 5–7 mice of each genotype (mean +/− standard deviation).

**Figure 6:**
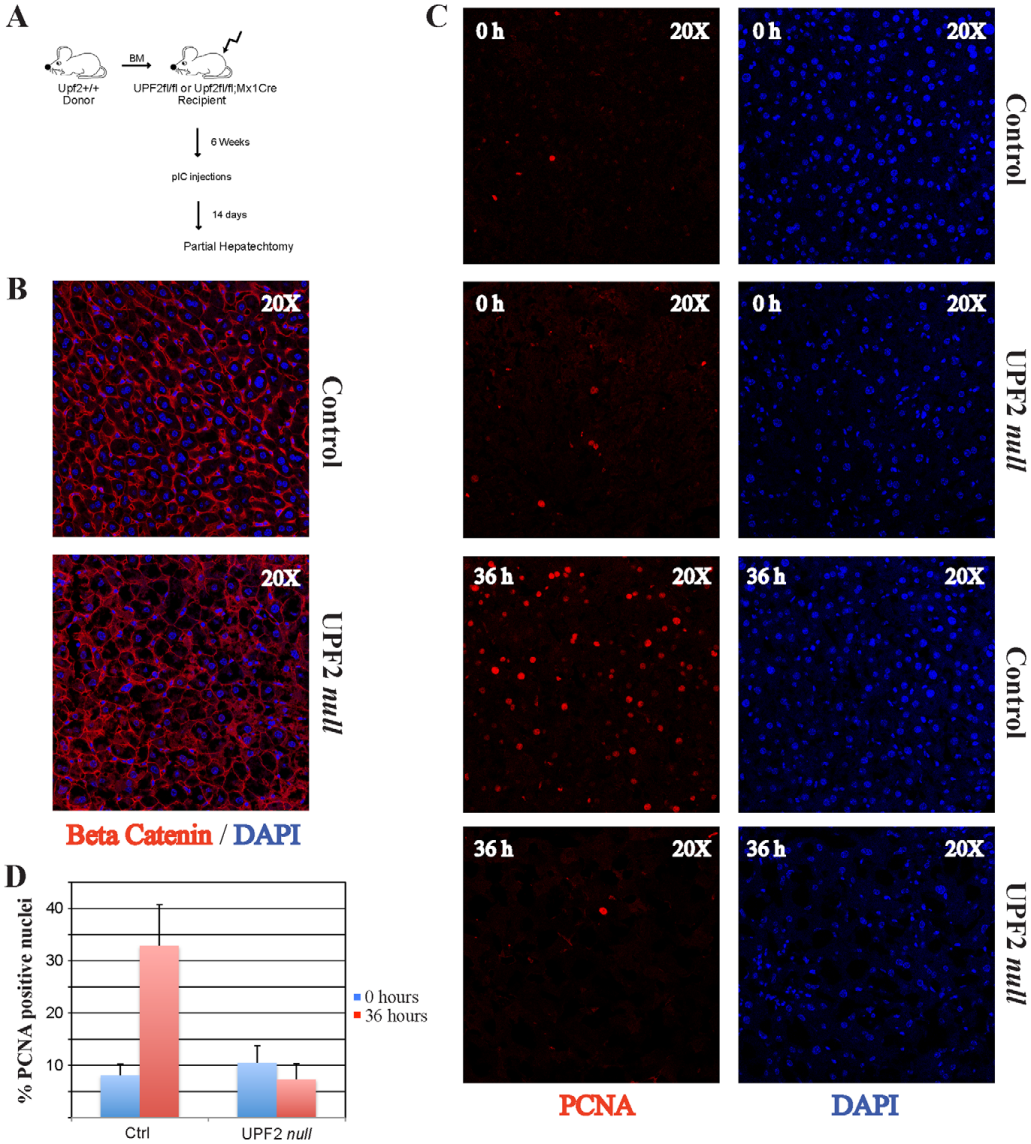
UPF2 is essential for liver regeneration. (A) Experimental strategy for the induction of UPF2 loss in the adult liver of BM transplanted control (*Upf2^fl/fl^*) and *Upf2^fl/fl^; Mx1Cre* mice followed by PH. (B) Immunofluorescence analysis of β-catenin of control and UPF2 *null* adult livers 14 days after the first pIC injection. (C) Immunofluorescence analysis of PCNA expression before (0 h) or after (36 h) PH. (D) Quantification of the data from (C), representing the average of three mice of each genotype at each time point. 200–500 nuclei were counted for each mouse at each time point (mean +/− standard deviation).

Having demonstrated that loss of UPF2 in the adult liver leads to liver damage, we next used gene expression profiling to probe the underlying cause. In order to avoid putative secondary effects caused by extensive tissue damage and invasion of WT immune cells observed at 21 days post deletion, we harvested livers from pIC injected BM transplanted *Upf2^fl/fl^* control and *Upf2^fl/fl^; Mx1Cre* experimental animals 14 days after initiating deletion of UPF2 (Figure 5A). At this time point livers from pIC injected *Upf2^fl/fl^; Mx1Cre* mice were fully recombined (Figure 5B), appeared essentially normal as assessed by histology (Figure 6B) and displayed no signs of infiltrating immune cells (Figure 5B). Gene expression profiling of RNA harvested from 14 days post deletion livers identified 1486 up-regulated (fold change >1.5; P<0.001) and 981 down-regulated probesets (fold change <−1.5; <0.001; Figure 5C,D; See Table S5 for full gene list). Again, we observed that loss of UPF2 leads to the up-regulation of 37 snoRNA host gene transcripts and that these transcripts are among the most up-regulated (Figure 5E, Table S2). As it has been demonstrated that NMD features may lead to nonsense-mediated transcriptional gene silencing (NMTGS), we next tested whether snoRNA host genes were transcribed at lower rates in UPF2 *null* livers [31]. Chromatin immunoprecipitation followed by qPCR (qChIP) showed that the gene bodies of 6 up-regulated snoRNA host genes were occupied by RNA polymerase II to the same degree in both WT and UPF2 *null* livers, suggesting that they are transcribed at similar rates in both genotypes (Figure 5F). Hence, these findings argues against a role of NMTGS in down-regulation of mature snoRNA host gene transcripts and demonstrate that these transcripts are indeed direct targets of the NMD machinery.

Similar to what we found in the fetal liver, GSEA (See Table S6 for the full analysis) revealed that loss of UPF2 in the adult setting led to down-regulation of genes involved in “HSA04610 Complement and Coagulation cascades” and metabolic pathways as exemplified by “Biosynthesis of Steroids” albeit at less significance as what resulted from embryonic loss of UPF2 (Figure 5G). Quantitative RT-PCR confirmed deregulation of these pathways (Figure 5H) and functional assessment of the complement system revealed a pronounced reduction in the activities of both the classical and alternative pathways in UPF2 *null* sera (Figure 5I, Figure S3). These findings demonstrate that hepatocytes require UPF2 for the generation of an efficient complement system.

GSEA also revealed similarity between fetal and adult livers among the gene sets for which expression correlated positively with loss of UPF2. Here, although no gene sets fell below our false discovery cut-off of 0.05, we did observe borderline up-regulation of gene sets in “mRNA processing reactome” and in the “Regulation of Mitosis” and we were able to validate the up-regulation of several of the leading edge transcripts by qRT-PCR (Figure 5G,H). As reported above, we could not detect any evidence for the induction of a DNA damage response above background in the adult UPF2 *null* livers – a finding entirely consistent with the failure of gene sets representing DNA damage to approach our significance cut-offs (data not shown).

Finally, we tested to what extent loss of UPF2 lead to the stabilization of known PTC+ mRNA isoforms derived from AS-NMD [9], [13]. Consistent with our previous findings in the hematopoietic system, loss of NMD promoted an increase in the relative abundance of the PTC+ isoform for 9/9 tested transcripts (Figure S4).

In summary, our findings demonstrate the vital importance of UPF2, and by inference NMD, in maintaining homeostasis of the adult liver.

### UPF2 deficient livers fail to regenerate following partial hepatectomy

We have previously demonstrated that deletion of UPF2 in the hematopoietic system led to a pronounced depletion of cycling progenitor cells in the BM ultimately resulting in BM failure [13]. In addition, UPF2 deleted lymphocytes were unable to enter the cell cycle upon stimulation, suggesting that UPF2 is required for active proliferating cells.

We took advantage of the regenerative power of the hepatectomized liver to test whether the loss of UPF2 was compatible with efficient regeneration. Specifically, we performed a 70% hepatectomy on BM transplanted *Upf2^fl/fl^* control and *Upf2^fl/fl^; Mx1Cre* mice two weeks after pIC injection and these mice were subsequently sacrificed 36 hours after surgery (Figure 6A). In contrast to control mice (BM transplanted *Upf2^fl/fl^*), PH in BM transplanted *Upf2^fl/fl^; Mx1Cre* mice was associated with a substantial post-surgery mortality (>50%), most likely reflecting the massive changes in the transcriptome of UPF2 deficient livers.

Immunofluorescence assessment of PCNA and Ki67 – both markers of actively cycling cells – revealed a marked difference between control and UPF2 *null* hepatocytes (Figure 6C,D, Figure S5). Whereas the former efficiently entered the cell cycle reaching a level of 32% PCNA positive nuclei 36 hours post PH, UPF2 deleted hepatocytes were essentially deficient in sustaining liver regeneration as assayed by both Ki67 and PCNA staining. Jointly, these data, and those from the hematopoietic system, unambiguously demonstrate that UPF2 is absolutely required for proliferating cells in both these tissues under relevant physiological conditions.

### Liver-specific loss of UPF2 interferes with the induction of a transcriptional program necessary for liver regeneration

**Figure 7:**
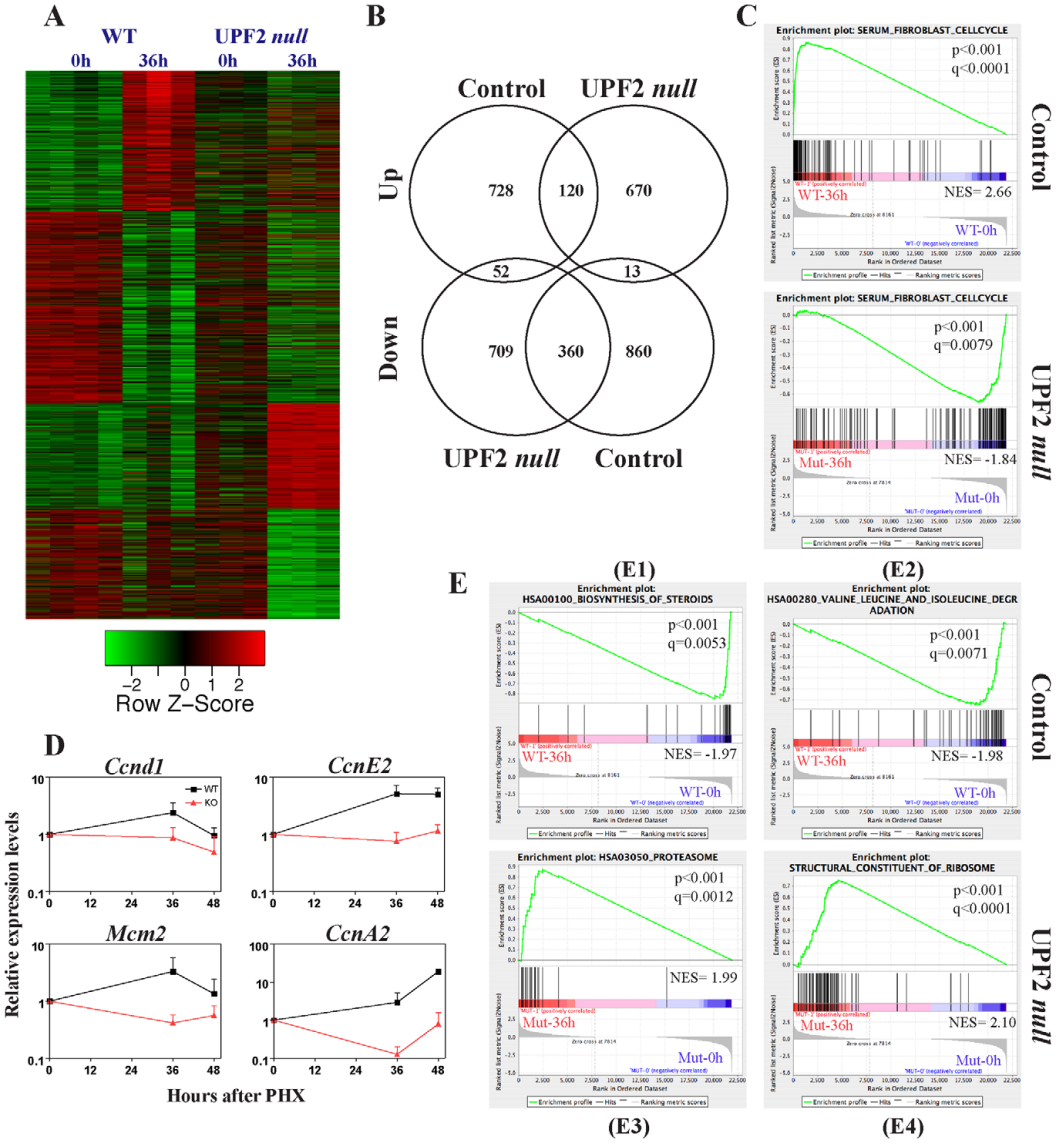
Gene expression analysis of UPF2 null adult livers following PH. (A) Cluster analysis of normalized affymetrix expression data from control and UPF2 *null* adult livers before (0 h) or after (36 h) PH. Venn diagrams of differentially expressed probesets following PH. The diagrams reveals the induction of partially overlapping gene expression programs in control and UPF2 *null* adult livers following PH. (C) GSEA reveals that gene sets involved in cell cycle regulation are differentially induced in control and UPF2 *null* adult liver following PH. (D) qRT-PCR validation of 4 cell cycle regulated and PH-induced genes. cDNA was synthesized from 3 mice of each genotype at each timepoint and assayed by qPCR in triplicates (mean +/− standard deviation). (E) Selected enriched gene sets from GSEA analysis of differentially expressed genes following PH in control and UPF2 *null* livers. E1: HSA00100_Biosynthesis_of_Steroids, E2: HSA00280_Valine_and_Isoleucine_degradation, E3: HSA03050_Proteasome, E4: Structural_Constituent_of_ribosome. Normalized enrichment scores, P-value and q-values (FDR) are indicated for each gene set. Negative and positive NES values indicate enrichment in control and UPF2 *null* adult livers, respectively. Y-axes indicate Enrichment scores (top) and Ranked list metric (bottom). Green lines indicate enrichment profile, black bars are hits (i.e. genes) and grey profiles are ranked metric score. Red and blue indicate positive and negative correlations, respectively, with the indicated gene sets.

UPF2 *null* hepatocytes are unable to re-enter the cell cycle following PH. In order to get insights into the underlying molecular cause for this phenotype, we next compared the gene expression changes induced by PH in control and UPF2 deficient livers. We adopted a similar protocol as described above in which BM transplanted *Upf2^fl/fl^* control and *Upf2^fl/fl^; Mx1Cre* mice were subjected to PH two weeks after pIC injection. Comparison of the gene expression profiles of control mice at surgery (0 hours) and 36 hours post-surgery revealed a massive transcriptional reprogramming induced by PH with 2123 probesets displaying significant changes (fold changes >1.5 or <−1.5; P<0.05, Figure 7A,B; See Table S7). PH of BM transplanted *Upf2^fl/fl^; Mx1Cre* animals also led to a pronounced transcriptional reprogramming with 1924 probesets (fold changes >1.5 or <−1.5; P<0.05) being changed when the 0 hours and 36 hours timepoints were compared. Despite the massive transcriptional reprogramming that occurs in both genotypes, only a limited fraction of the changes are shared by control and mutant livers (Figure 7B). Among the down-regulated probesets 29–32% (360/1233 in control and 360/1121 in mutant) are common for the two genotypes, whereas the corresponding fraction for up-regulated probesets are as low 13–15% (120/900 in control and 120/803 in mutant).

We next employed GSEA on the 4 data sets (up and down-regulated genes for each of the two genotypes) with the aim to identify gene programs specific for the two genotypes. Entirely consistent with the phenotypic data, we found that whereas control livers up-regulate a series of gene sets associated with active cell cycle (e.g. “Serum_Fibroblast_CellCycle” in Figure 7C, See Table S8 for full analysis) following PH, UPF2 deficient livers fail to do so. Actually, PH following loss of UPF2 appears to lead to a further withdrawal from the cell cycle as suggested by the down-regulation of gene sets associated with active cell cycle (Figure 7C). We validated these findings by qRT-PCR on four cell cycle regulated and PH-induced transcripts (*Ccnd1*, *CcnE2*, *Mcm2*, *CcnA2*) and found that all four are induced in control mice and down-regulated in animals lacking UPF2 in the liver (Figure 7D). The finding that none of these four transcripts are induced in mutant livers even at 48 hours after surgery argues against a delayed cell cycle entry caused by loss of UPF2.

GSEA further showed that whereas PH in control livers induced a dedifferentiation program at 36 hours as evidenced by down-regulation of metabolic and catabolic gene sets (as exemplified by “HSA001000_Biosynthesis_of_steroids” and HSA00280_Valine_Leucine_and_Isoleucine_Degradation in Figure 7E), none of these gene sets were significantly enriched in UPF2 deficient livers (Table S8). In contrast, UPF2 deficient livers respond to PH by up-regulating gene sets associated with proteasome and ribosome function (as exemplified by “HSA03050_Proteasome” and “Structural_Constituent_Of_Ribosome” (Figure 7E)) perhaps reflecting a stage where the regenerating liver is preparing for increased cellular turnover. Furthermore, the similar levels of *Socs3* and *Saa2* induction (downstream STAT3 targets) at 36 hours post PH, suggest that activation of the STAT pathway during the priming phase of PH has occurred normally in mutant livers (Table S3). Similarly, the expression of both *Cebpa* and *Cebpb*, two transcription factors repressed and induced, respectively, during the priming phase of PH, also appear comparable in control and UPF2 *null* livers.

Collectively our analysis of the transcriptional response to PH reveals that lack of UPF2 leads to a block in liver regeneration at a stage before dedifferentiation and entry into the cell cycle and further underlines the strong requirement for UPF2 in proliferating cells.

## Discussion

The NMD pathway is a critical posttranscriptional pathway regulating mRNA stability by its ability to find and destroy PTC+ mRNAs. NMD targets are highly divergent, both in terms of functional classes, but also due to their poor lack of evolutionary conservation. Thus there is an urgent need to develop appropriate experimental models that allow us to test the functional role of NMD in mammalian systems relevant for human biology and disease.

Here we report on the first *in vivo* characterization of the importance of RNA surveillance at several stages of the life cycle of the liver including development, homeostasis and regeneration. The present data demonstrate that loss of UPF2, and by inference NMD, affects all these stages through several distinct mechanisms.

### NMD and Liver function

During embryonic development the fetal liver plays pivotal roles both in the preparation for postnatal life but also as the primary hematopoietic organ *in utero*. Loss of UPF2, initiated from the early stages of hepatoblast migration and onwards, resulted in relative minor phenotypes, the main being the lack of terminal differentiation of fetal liver cells at a stage beyond the HNF4α-dependent epithelial transformation. The inability of UPF2 *null* fetal livers to undergo terminal differentiation was incompatible with postnatal life. The relatively normal fetal liver development of UPF2 *null* livers is further supported by the lack of anemia in these embryos (data not shown), which would be predicted if the liver experienced gross developmental abnormalities. A formal possibility for the mild phenotype could be partial hepatoblast-specific deletion of UPF2. However, this is not consistent with our gene expression analysis demonstrating down-regulation of several transcripts to background levels (Table S1).

In contrast to the relatively minor phenotype of UPF2 *null* fetal livers, induced loss of UPF2 is highly detrimental to a steady-state adult liver. Thus, within 3 weeks post deletion the liver has deteriorated functionally as evidenced by down-regulation of metabolic transcriptional programs, hepatosteatosis and signs of liver damage. This is accompanied by hepatosteatosis, i.e. accumulation of lipids in the liver, presumably caused by a reduced capacity of the organ to metabolize lipids. The functional deterioration of the liver, which is also evidenced by increased serum levels of bilirubin, occurs in the absence of detectable apoptosis. Furthermore, when UPF2 *null* liver is subjected to PH two weeks post deletion, hepatocytes arrest at a stage past the priming phase but prior to entering active cell cycle.

Gene expression analysis before and after PH show that UPF2 *null* mice induce a transcriptional program that overlap only partially with the one induced in WT mice and suggest that the NMD pathway may modulate central components required for regeneration. Interestingly, loss of UPF2 leads to a selective down-regulation of transcripts associated with complement activity including *C1S, C2* and *C8B* in both fetal and in steady state adult liver; and importantly, that this down-regulation was associated with reductions in both the classical and alternative complement pathways. This result is particular intriguing in light of the inability of livers deficient for the complement factors C3 or C5 to regenerate in response to PH [25]. Thus, down-regulation of complement activity, along with the requirement of NMD for active cell cycling (see below), suggest molecular mechanisms by which regeneration is blocked in UPF2 deficient mice.

### NMD and the Cell cycle

Tissue-culture studies have previously implicated NMD factors in cell cycle progression in mammalian cells. These include UPF1, SMG1 and SMG6, whereas UPF2 was reported to be dispensable [12], [18], [19]. The latter result contrast findings from *Drosophila* cell lines and our own analysis of UPF2 deficient lymphocytes where loss of UPF2 in both cases interfered with cellular proliferation [13], [32]. Consistently, we find that deletion of UPF2 results in a complete lack of recruitment into active cell cycling following PH. At present, we have not pinpointed the cell cycle defects to any distinct phase of the cell cycle, however, preliminary data from UPF2 *null* lymphocytes suggest that loss of NMD in these cells results in an early block in S-phase (JW and BTP, unpublished observations). UPF2 *null* fetal livers also display a phenotype consistent with defects in normal cell cycle progression, albeit not to the same extent as during PH, with accumulation of abnormal mitotic cells. Furthermore, the accumulation of a marker for double-stranded DNA breaks in UPF2 *null* hepatoblast is compatible with problems transversing the S-phase in these mice. This notion is further corroborated by the fact that regenerating UPF2 *null* adult hepatocytes, which are completely unable to proliferate, also lack any detectable activation of DNA damage pathways, suggesting that this activation is associated with active proliferation. In any event, our findings further reinforce the requirement of NMD components, including UPF2, for active proliferation in mammalian cells although our data from the fetal liver shows that there are important cell type- and/or developmental-specific differences. As loss of several individual NMD components in tissue-culture converge at blocking cell cycle progression, this most likely reflects the activity of the NMD pathway *per se* and not any non-NMD functions of individual members. This view is consistent with recent studies from zebrafish, where knockdown of individual NMD components yielded highly similar phenotypes suggesting that they are conferred by ablation of NMD function as such [33].

As the NMD pathway affects both mRNA isoform distributions as well as transcript levels, unraveling the underlying molecular mechanism(s) for the NMD requirement in proliferation will require full whole-transcriptome mapping studies of NMD deficient mammalian cells as well as the development of a conditional NMD *null* cell system amenable to rescue experiments. Such experiments are currently underway.

### NMD and gene regulation

The NMD pathway regulates two main classes of transcripts: The first class encompasses transcripts, which have undergone erroneous processing leading to the generation of translatable PTC+ variants fulfilling the molecular rules of NMD. The second class of transcripts is non-erroneous and gives rise to NMD by a variety of mechanisms [12], [13], [34]. For these transcripts, NMD serves as an important posttranscriptional regulator of transcript levels.

Our gene set enrichment analysis suggested that NMD is essential for the proper regulation of selected transcript classes. In particular, we find that transcripts belonging to the “mRNA processing” category, including regulators of splicing, are significantly up-regulated upon loss of UPF2. This is particular intriguing in light of the established role of NMD in setting up autoregulatory loops through AS-NMD. Hence our data suggest that disturbing these feedback mechanisms lead to the deregulation of transcript levels of a subset of these factors, perhaps through expression of dominant negative variants. As splice site selection is believed to be controlled by a competition between positive and negative regulators, such as SR (Serine-Arginine rich) and hnRNP proteins [35], [36], changing the relative levels of these splicing modulators may have profound overall consequences for global splicing patterns. Future whole-transcriptome experiments of relevant UPF2 deficient tissues will formally test this possibility.

SnoRNA host genes constitute a diverse class of transcripts which harbor snoRNA genes within their intronic sequences [37]. SnoRNA coding sequences can reside in either protein coding genes or in pseudogenes, which have lost their protein coding potential while maintaining their exon-intron structures, perhaps to facilitate efficient snoRNA expression. These small RNAs are involved in guiding functional important posttranscriptional modifications of cellular RNAs, mainly rRNAs, and are therefore often abundantly expressed. Here we show that more than 40 snoRNA host genes are regulated by NMD in at least one tissue. Furthermore, as loss of NMD does not result in any differences in RNA Polymerase II occupancy for 6/6 tested highly up-regulated snoRNA host genes, our data imply that snoRNA host genes are normally efficiently degraded in the presence of a functional NMD pathway. We conclude that NMD has evolved as an efficient regulator of snoRNA metabolism perhaps to allow the abundant expression of a subset of these RNAs.

In addition to the more special cases of splicing regulators and snoRNA host genes, the NMD pathway also regulate a range of other transcripts. There may be several reasons as to why the cell chooses to express transcripts with obvious NMD features such as uORFs and introns within the 3′UTR. One possible explanation is offered by the observation that NMD occurs with different efficiencies in different cell types and may therefore be involved in generating tissue-specific expression patterns, in a manner reminiscent of miRNAs [14]. It also remains possible that NMD efficiency could be regulated by intra- and extracellular cues, perhaps through posttranscriptional modifications of key NMD factors. This would allow the cell to either up-regulate or down-regulate the transcript levels of NMD targets in response to different stimuli. These two possibilities do by no means exclude each other.

In summary, our data provides novel insights into the importance of RNA surveillance in general, and NMD in particular, for mammalian biology. We have shown that the loss of NMD at different phases of the life cycle of the liver has distinct phenotypic consequences, suggesting that NMD plays multiple roles in liver function. These include differential requirements for NMD in proliferating cells, regulation of distinct transcript classes as well as more systemic effects on alternative splicing. As different cell types may have different needs, our studies together with those from our previous analysis of the hematopoietic system [13], highlights the importance of characterizing general systems, such as the NMD pathway, in several different tissues.

## Materials and Methods

### Ethics statement

All mouse work was performed according to national and international guidelines and approved by the Danish Animal Ethical Committee. This study was approved by the review board at the Faculty of Health, University of Copenhagen (LT-P0658).

### Mice strains and Procedures

The generation and genotyping of UPF2 conditional knockout mouse has been reported previously [13]. The conditional *Upf2^fl/+^* allele was crossed onto the tissue-specific *Alfp-Cre* [27] and Mx1-Cre [30] deleter strains. All experimental animals had been backcrossed to the C57/B6 background for at least six generations.

For the fetal liver knockout of UPF2, *Upf2^fl/+^; Alfp-Cre* males were crossed with *Upf2^fl/+^* females and embryos were harvested from pregnant females at E13.5, E16.5 and E18.5. For determination of fetal liver proliferation, pregnant females received a single intraperitoneal injection of 5-bromo-2-deoxyuridine (100 µg BrdU/g body weight; Sigma) prior to sacrifice. The sex of the embryos was determined using primers specific for the *Sry* gene located at the Y chromosome. For the generation of the adult liver-specific knockout of UPF2, 10–12 weeks old female *Upf2^fl/fl^* and *Upf2^fl/fl^; Mx1Cre* animals (CD45.2) were lethally irradiated by a single radiation dose (900 cGY) and transplanted with 5 million BM cells from CD45.1 C57/B6. Six weeks after transplantation recipient mice were injected 3 times (Day 0, Day 2 and Day 4) with pIC (300 µg, GE Healthcare). Mice were either sacrificed (at day 14 or day 21) or subjected to PH.

Partial hepatectomy was performed by removal of 70% of the liver mass (i.e. the median and left lateral lobes) under Isoflurane inhalation anaesthesia. A midline abdominal skin and muscle incision of 1.5 cm was used to expose the Xiphoid process. The lobes were tied off separately and excised while keeping the gall bladder and suprahepatic vena cava intact. Subsequently, the peritoneum and the skin were sutured and the animals left to recover. Hepatectomized animals were sacrificed at 36 hours, 48 hours or 72 hours after the procedure.

### Immunohistochemistry and immunofluorescence microscopy

Livers were harvested, washed in PBS and subjected to four overnight incubations at 4°C. Day 1 in 4% PFA, day 2 in 30% sucrose, day 3 in 1∶1 mix of 30% sucrose and OCT (VWR), day 4 in OCT only. On day 5 livers were embedded with OCT in cryomolds and stored at -80°C. Livers were cryo-sectioned (6–8 µm) onto Tonto SuperFrost® microscope slides (VWR) and stored at −80°C. Antigen retrieval was obtained by boiling in 0.01 M, pH 6.0 citrate buffer for 10 min. followed by cooling to room temperature. Slides were blocked with 5% BSA in PBS and subsequently stained with primary mouse monoclonal antibodies against Ki67 (clone B56; BD Biosciences Pharmingen, San Jose, CA, USA), PCNA (clone PC10; Santa Cruz Biotechnology, Santa Cruz, CA, USA), β-catenin (clone 14; Transduction laboratories, Lexington, Kentucky, USA) phospho-S139-γH2AX (clone JBW301; Millipore), BrdU (clone Bu20a, DAKO) or rabbit polyclonal antibodies against phosho-Ser10-HH3 (Millipore) and Albumin (DAKO, Copenhagen, Denmark) over night at 4°C. Slides were the incubated with relevant secondary antibodies for 1 hour, i.e. either secondary goat-anti-mouse Alexa Fluor® 594 or goat-anti-rabbit Alexa Fluor® 594 antibodies (Invitrogen, Eugene, Oregon, USA) or using the Envision+ system (Dako, Denmark) according to the manufacturers instructions. Nuclei were stained with DAPI (Invitrogen) at 1 µg/mL for 5 min. Sections were examined using either a Zeiss Axioplan2 imaging fluorescence microscope, a Leica TCS SP2 confocal microscope, or a Olympus BX51d microscope.

Hematoxylin-eosin (HE) staining of fetal and adult liver sections was performed as described previously [38]. For the staining of livers for accumulation of lipids fixed slides was incubated 8 min. in pre-warmed (60°C) Oil Red O solution (0.5% in propylene glycol), destained 2×5 min in propylene glycol and rinsed twice in water. TUNEL (Terminal deoxynucleotidyl transferase-mediated biotinylated UTP nick end labeling) was performed using an *In situ* Cell Death Detection Kit (Roche) according to the manufacturers instructions.

### Immunoprecipitation

Liver lysates were prepared by homogenizing liver in ice-cold RIPA buffer (20 mM Tris-HCl, pH 7.2, 1% sodium deoxycholate, 1% Triton X-100, 0.1% SDS, 150 mM NaCl plus 2 mM PMSF, 2 µg/mL aprotinin, 2 µg/mL leupeptin) with a loose-pestle Dounce homogenizer on ice for 10 min, resulting in a 50% (w/w) liver homogenate. The homogenates were incubated on ice for 30 min and clarified by centrifugation (14,000×*g*; 30 min; 4°C) and the resulting lysates were used immediately or stored at −80°C.

To immunoprecipitate UPF2, the lysates were incubated with Protein G agarose-bound affinity purified rabbit anti-UPF2 polyclonal antibody (the antibody was a kind gift from Dr Jens Lykke-Andersen) by rotating at 4°C overnight. Next, the UPF2-bound agarose slurry was spun down and the supernatant was used to precipitate alpha-Tubulin, using mouse monoclonal anti-Tubulin antibody (T9026, Sigma) bound to Protein A/G. After binding, the protein-bound slurries were washed three times, resuspended in 2× SDS Loading buffer and boiled. Proteins were separated by SDS-PAGE followed by Western blotting, using a Mini-Trans Blot Electrophoretic Transfer Cell (BioRad).

### Chromatin immunoprecipitation

Livers were harvested, directly snap-frozen in liquid nitrogen and stored at −80°C. After thawing, tissue samples were homogenized by douncing (loose pestle, Wheaton 15 ml douncer) in cold PBS, and cross-linked for 10 min. in 1% formaldehyde using a rotator. ChIP was performed using 30 µg chromatin to 2 µl of POL-II subunit B1 antibody (AC-055-100, Diagenode) with washing steps and DNA retrieval as described previously [39]. Enrichment ratios were examined by qPCR (ABI Prism 7000) with primer sets directed against internal gene body positions of the indicated genes versus a negative position at a gene desert on chromosome 12. Data are given as enrichment ratios normalized to the negative detector and further expressed relative to those of *Actb*.

### Quantitative RT-PCR analysis

cDNA was synthesized using Protoscript First Strand cDNA Synthesis Kit (New England Biolabs), according to manufacturer's instructions. Briefly, oligo dT primer was used to prime first-strand cDNA synthesis from 0.5 ug of total RNA using dNTP mix, 1× RT Buffer, RNase inhibitor and M-MuLV Reverse Transcriptase at 42°C for 1 h. Finally, the RNA templates were degraded, by treating with RNase H at 37°C for 20 min.

The single-stranded cDNA was diluted 1∶10 and analyzed by qPCR using 0.3 µM primers (see Table S9 for list of primers), with Maxima SYBR Green qPCR Master Mix (Fermentas), as described by the manufacturer. The qPCR was run on a 7000 Sequence Detection System (Applied Biosystems) and analyzed with the accompanying software. Expression values are normalized to *Rrpo*.

For the detection of alternative splicing, cDNA was used as template for standard PCR reactions using primers against selected mRNA isoforms (Table S9).

### Serum analysis

Peripheral blood was collected into Microtainer tubes containing lithium heparin gel (BD). Samples were spun down at 2000 g for 10 min. after which serum was collected into clean eppendorf tubes and stored at −20°C. Samples were analyzed on a Modular (Roche Diagnostics).

### Complement activity

Peripheral blood was allowed to clot for 20 min. at room temperature, followed by 15 min. incubation on ice and centrifugation for 15 min. at 7500 g. Serum was withdrawn and stored at −80°C. Activity of the classical pathway was determined from C3 deposition on K562 cells (ATCC, LGC Promochem, Boras, Sweden) opsonised with affinity-purified rabbit polyclonal antibodies raised against K562 cells. Briefly, K562 cells were washed twice in cold PBS and 500,000 cells were added to reaction tubes containing PBS with 2 mM MgCl_2_, 0.15 mM CaCl_2_, 5 mg/ml of antibodies and 10% mouse sera in a total volume of 50 µl. After 30 min incubation at 37°C cells were washed with cold PBS with 1% fetal calf serum. The amount of deposited C3b was measured using FITC-conjugated goat anti-mouse C3 (ICN/MP Biomedicals, Illkirch, France) diluted 1∶100 and allowed to bind for 1 h at 4°C. The cells were washed three times with cold PBS with 1% fetal calf serum and analyzed by flow cytometry. Activity of the alternative pathway was determined using a hemolytic assay of rabbit erythrocytes [40].

### RNA isolation and gene expression profiling

Livers were snap-frozen in liquid nitrogen immediately after harvest. Small tissue slices were homogenized in Trizol using a Pellet Pestle Motor (Kontes). Total RNA was isolated according to the manufactures protocol (Invitrogen).

Two independent microarray analyses were performed using fetal liver and adult liver (before and after PH). RNA samples were labeled as described previously [13] and hybridized to the Mouse Genome 430 2.0 GeneChip (Affymetrix, Santa Clara, CA, USA). Cel files were imported into the statistical software package R v. 2.7.2 using BioConductor [41] and gcRMA modeled using quantiles normalization and “lowess” summarization [42]. Detection calls were determined by the MAS 5.0 algorithm implemented in the Bioconductor package affy (100% present calls required within each replicate group) and the modeled log-intensities of the probe sets were used for high-level analysis of selecting differentially expressed genes.

For the fetal liver E16.5 and E18.5 data sets, control and mutant replicate groups were compared and genes were defined to be differentially expressed if they were up or down regulated with a fold change above 2 and a p-value below 0.05 (paired Welch t-test).

For the adult liver data sets, genes were defined to be differentially expressed if they were up or down-regulated with a fold change above 1.5, a difference of means (absolute change) above 75 (unlogged expression values) and Benjamini Hochberg corrected p-values below 0.05 (Students t-test using log2 expression values) for samples undergoing PH or 0.001 for the comparison between control and mutant prior to PH. All microarray data are in compliance with MIAME and were deposited in ArrayExpress (E-MEXP-2248 and E-MEXP-2249). Hierarchical cluster heatmaps were generated from relevant subset of genes in R (v.2.10.0) using complete linkage and Pearson correlation distance measure.

Gene sets enrichment analyses were performed using the GSEA software v2.04 and gene sets from the MSigDB available at www.broad.mit.edu/gsea (permutation type gene sets, 1000 permutations). Enrichments plots from the GSEA program are shown along with normalized enrichment scores (NES), P-values and false detection rate q value for the corresponding gene sets [28]. q values <0.05 are considered significant.

## Supporting Information

Figure S1 Loss of UPF2 during fetal liver development does not affect proliferation rates, number of mitotic cells or apoptosis. (A) Histological analysis of control (Upf2fl/f) and UPF2 null (Upf2fl/fl; Alfp-Cre) E16.5 fetal livers stained with HE. (B) E18.5 UPF2 null fetal livers incorporate BrdU to similar extent as control livers. (C) E16.5 UPF2 null fetal livers have similar number of mitotic cells as assayed by phosphorylation of S10 of Histone H3. (D) TUNEL staining of E16.5 UPF2 null and control fetal livers.
(5.38 MB TIF)

Figure S2 Loss of UPF2 in adult liver does not induce apoptosis. TUNEL staining of adult control and UPF2 null livers harvested 21 days post deletion.
(1.77 MB TIF)

Figure S3 Loss of UPF2 functionally compromises the classical complement pathway. FACS analysis of C3b deposition on K562 opsonised with antibodies against K562 using sera from control (Upf2fl/fl) and UPF2 null (Upf2fl/fl;Mx1Cre) mice. Serum was harvested 2 weeks post deletion of UPF2.
(0.20 MB TIF)

Figure S4 Loss of UPF2 in adult liver leads to altered splicing patterns. RT-PCR analysis of RNA isolated from adult control (Upf2fl/fl) and UPF2 null (Upf2fl/fl; Mx1Cre) livers shows that PTC-containing alternative spliced isoforms were stabilized for 9/9 tested genes. Up-regulated splice isoforms are indicated by an asterisk.
(0.49 MB TIF)

Figure S5 UPF2 is essential for liver regeneration. Immunofluorescence analysis of Ki67 expression before (0 h) or after (36 h) PH of control and UPF2null BM transplanted mice.
(2.18 MB TIF)

Table S1 Deregulated probesets in Upf2fl/fl; AlfpCre E16.5 and E18.5 fetal livers. Probesets displaying expression fold changes >2.0 or <−2.0 (paired Welsch test P<0.05) are depicted.
(6.18 MB XLS)

Table S2 SnoRNAs host genes and resident SnoRNAs. SnoRNA host genes showing up-regulation in the indicated tissues upon UPF2 truncation are depicted.
(0.08 MB DOC)

Table S3 Average affymetrix gene expression fold changes for selected genes. In case of multiple probesets the set with the highest expression levels are shown.
(0.04 MB DOC)

Table S4 GSEA of normalized gene expression datasets derived from control and UPF2 null fetal livers at E16.5 and E18.5. The top 100 up- and down-regulated gene sets are depicted for both timepoints. See Supplemental Table S1 for lists of up- and down-regulated probesets.
(9.66 MB XLS)

Table S5 Deregulated probesets in adult UPF2 null livers. Probesets displaying expression fold changes >1.5 or <−1.5 (P<0.001) are depicted.
(27.02 MB XLS)

Table S6 GSEA of normalized gene expression datasets derived from control and UPF2 null adult livers. The top 100 up- and down-regulated gene sets are depicted. See Table S5 for lists of up- and down-regulated probesets.
(0.06 MB XLS)

Table S7 Deregulated probesets in adult control and/or UPF2 null livers following PH. Probesets displaying expression fold changes >1.5 or <−1.5 (P<0.05) are depicted.
(2.71 MB XLS)

Table S8 GSEA of normalized gene expression datasets derived from control and UPF2 null adult livers following PH. The top 100 up- and down-regulated gene sets are depicted for phenotypes. See Table S7 for lists of up- and down-regulated probesets.
(0.10 MB XLS)

Table S9 Primers used in this study.
(0.07 MB DOC)

## References

[1] Rebbapragada I, Lykke-Andersen J (2009). Execution of nonsense-mediated mRNA decay: what defines a substrate?. Curr Opin Cell Biol.

[2] Isken O, Maquat LE (2007). Quality control of eukaryotic mRNA: safeguarding cells from abnormal mRNA function.. Genes Dev.

[3] Le Hir H, Gatfield D, Izaurralde E, Moore MJ (2001). The exon-exon junction complex provides a binding platform for factors involved in mRNA export and nonsense-mediated mRNA decay.. Embo J.

[4] Singh G, Rebbapragada I, Lykke-Andersen J (2008). A competition between stimulators and antagonists of Upf complex recruitment governs human nonsense-mediated mRNA decay.. PLoS Biol.

[5] Eberle AB, Stalder L, Mathys H, Orozco RZ, Muhlemann O (2008). Posttranscriptional gene regulation by spatial rearrangement of the 3′ untranslated region.. PLoS Biol.

[6] Buhler M, Steiner S, Mohn F, Paillusson A, Muhlemann O (2006). EJC-independent degradation of nonsense immunoglobulin-mu mRNA depends on 3′ UTR length.. Nat Struct Mol Biol.

[7] Weischenfeldt J, Lykke-Andersen J, Porse B (2005). Messenger RNA surveillance: neutralizing natural nonsense.. Curr Biol.

[8] Chang YF, Imam JS, Wilkinson MF (2007). The nonsense-mediated decay RNA surveillance pathway.. Annu Rev Biochem.

[9] Ni JZ, Grate L, Donohue JP, Preston C, Nobida N (2007). Ultraconserved elements are associated with homeostatic control of splicing regulators by alternative splicing and nonsense-mediated decay.. Genes Dev.

[10] Lareau LF, Inada M, Green RE, Wengrod JC, Brenner SE (2007). Unproductive splicing of SR genes associated with highly conserved and ultraconserved DNA elements.. Nature.

[11] Green RE, Lewis BP, Hillman RT, Blanchette M, Lareau LF (2003). Widespread predicted nonsense-mediated mRNA decay of alternatively-spliced transcripts of human normal and disease genes.. Bioinformatics.

[12] Wittmann J, Hol EM, Jack HM (2006). hUPF2 silencing identifies physiologic substrates of mammalian nonsense-mediated mRNA decay.. Mol Cell Biol.

[13] Weischenfeldt J, Damgaard I, Bryder D, Theilgaard-Monch K, Thoren LA (2008). NMD is essential for hematopoietic stem and progenitor cells and for eliminating by-products of programmed DNA rearrangements.. Genes Dev.

[14] Zetoune AB, Fontaniere S, Magnin D, Anczukow O, Buisson M (2008). Comparison of nonsense-mediated mRNA decay efficiency in various murine tissues.. BMC Genet.

[15] Brumbaugh KM, Otterness DM, Geisen C, Oliveira V, Brognard J (2004). The mRNA surveillance protein hSMG-1 functions in genotoxic stress response pathways in mammalian cells.. Mol Cell.

[16] Yamashita A, Izumi N, Kashima I, Ohnishi T, Saari B (2009). SMG-8 and SMG-9, two novel subunits of the SMG-1 complex, regulate remodeling of the mRNA surveillance complex during nonsense-mediated mRNA decay.. Genes Dev.

[17] Eberle AB, Lykke-Andersen S, Muhlemann O, Jensen TH (2009). SMG6 promotes endonucleolytic cleavage of nonsense mRNA in human cells.. Nat Struct Mol Biol.

[18] Azzalin CM, Lingner J (2006). The double life of UPF1 in RNA and DNA stability pathways.. Cell Cycle.

[19] Azzalin CM, Lingner J (2006). The human RNA surveillance factor UPF1 is required for S phase progression and genome stability.. Curr Biol.

[20] Azzalin CM, Reichenbach P, Khoriauli L, Giulotto E, Lingner J (2007). Telomeric repeat containing RNA and RNA surveillance factors at mammalian chromosome ends.. Science.

[21] Medghalchi SM, Frischmeyer PA, Mendell JT, Kelly AG, Lawler AM (2001). Rent1, a trans-effector of nonsense-mediated mRNA decay, is essential for mammalian embryonic viability.. Hum Mol Genet.

[22] Zaret KS, Grompe M (2008). Generation and regeneration of cells of the liver and pancreas.. Science.

[23] Battle MA, Konopka G, Parviz F, Gaggl AL, Yang C (2006). Hepatocyte nuclear factor 4alpha orchestrates expression of cell adhesion proteins during the epithelial transformation of the developing liver.. Proc Natl Acad Sci U S A.

[24] Cressman DE, Greenbaum LE, DeAngelis RA, Ciliberto G, Furth EE (1996). Liver failure and defective hepatocyte regeneration in interleukin-6-deficient mice.. Science.

[25] Strey CW, Markiewski M, Mastellos D, Tudoran R, Spruce LA (2003). The proinflammatory mediators C3a and C5a are essential for liver regeneration.. J Exp Med.

[26] Riehle KJ, Campbell JS, McMahan RS, Johnson MM, Beyer RP (2008). Regulation of liver regeneration and hepatocarcinogenesis by suppressor of cytokine signaling 3.. J Exp Med.

[27] Kellendonk C, Opherk C, Anlag K, Schutz G, Tronche F (2000). Hepatocyte-specific expression of Cre recombinase.. Genesis.

[28] Subramanian A, Tamayo P, Mootha VK, Mukherjee S, Ebert BL (2005). Gene set enrichment analysis: a knowledge-based approach for interpreting genome-wide expression profiles.. Proc Natl Acad Sci U S A.

[29] Saltzman AL, Kim YK, Pan Q, Fagnani MM, Maquat LE (2008). Regulation of multiple core spliceosomal proteins by alternative splicing-coupled nonsense-mediated mRNA decay.. Mol Cell Biol.

[30] Kuhn R, Schwenk F, Aguet M, Rajewsky K (1995). Inducible gene targeting in mice.. Science.

[31] Buhler M, Mohn F, Stalder L, Muhlemann O (2005). Transcriptional silencing of nonsense codon-containing immunoglobulin minigenes.. Mol Cell.

[32] Rehwinkel J, Letunic I, Raes J, Bork P, Izaurralde E (2005). Nonsense-mediated mRNA decay factors act in concert to regulate common mRNA targets.. Rna.

[33] Wittkopp N, Huntzinger E, Weiler C, Sauliere J, Schmidt S (2009). Nonsense-mediated mRNA decay effectors are essential for zebrafish embryonic development and survival.. Mol Cell Biol.

[34] Mendell JT, Sharifi NA, Meyers JL, Martinez-Murillo F, Dietz HC (2004). Nonsense surveillance regulates expression of diverse classes of mammalian transcripts and mutes genomic noise.. Nat Genet.

[35] Zhu J, Mayeda A, Krainer AR (2001). Exon identity established through differential antagonism between exonic splicing silencer-bound hnRNP A1 and enhancer-bound SR proteins.. Mol Cell.

[36] Martinez-Contreras R, Cloutier P, Shkreta L, Fisette JF, Revil T (2007). hnRNP proteins and splicing control.. Adv Exp Med Biol.

[37] Kiss T, Fayet E, Jady BE, Richard P, Weber M (2006). Biogenesis and intranuclear trafficking of human box C/D and H/ACA RNPs.. Cold Spring Harb Symp Quant Biol.

[38] Hasemann MS, Damgaard I, Schuster MB, Theilgaard-Monch K, Sorensen AB (2008). Mutation of C/EBP{alpha} predisposes to the development of myeloid leukemia in a retroviral insertional mutagenesis screen.. Blood.

[39] Sandmann T, Jakobsen JS, Furlong EE (2006). ChIP-on-chip protocol for genome-wide analysis of transcription factor binding in Drosophila melanogaster embryos.. Nat Protoc.

[40] Lindqvist AK, Johannesson M, Johansson AC, Nandakumar KS, Blom AM (2006). Backcross and partial advanced intercross analysis of nonobese diabetic gene-mediated effects on collagen-induced arthritis reveals an interactive effect by two major loci.. J Immunol.

[41] Gentleman RC, Carey VJ, Bates DM, Bolstad B, Dettling M (2004). Bioconductor: open software development for computational biology and bioinformatics.. Genome Biol.

[42] Bolstad BM, Irizarry RA, Astrand M, Speed TP (2003). A comparison of normalization methods for high density oligonucleotide array data based on variance and bias.. Bioinformatics.

