# Supplementary figures and images for: UPF2 Is a Critical Regulator of Liver Development, Function and Regeneration
Source: PLoS One. 2010 Jul 19;5(7):e11650. doi: 10.1371/journal.pone.0011650 (PMC2906512; doi:10.1371/journal.pone.0011650)

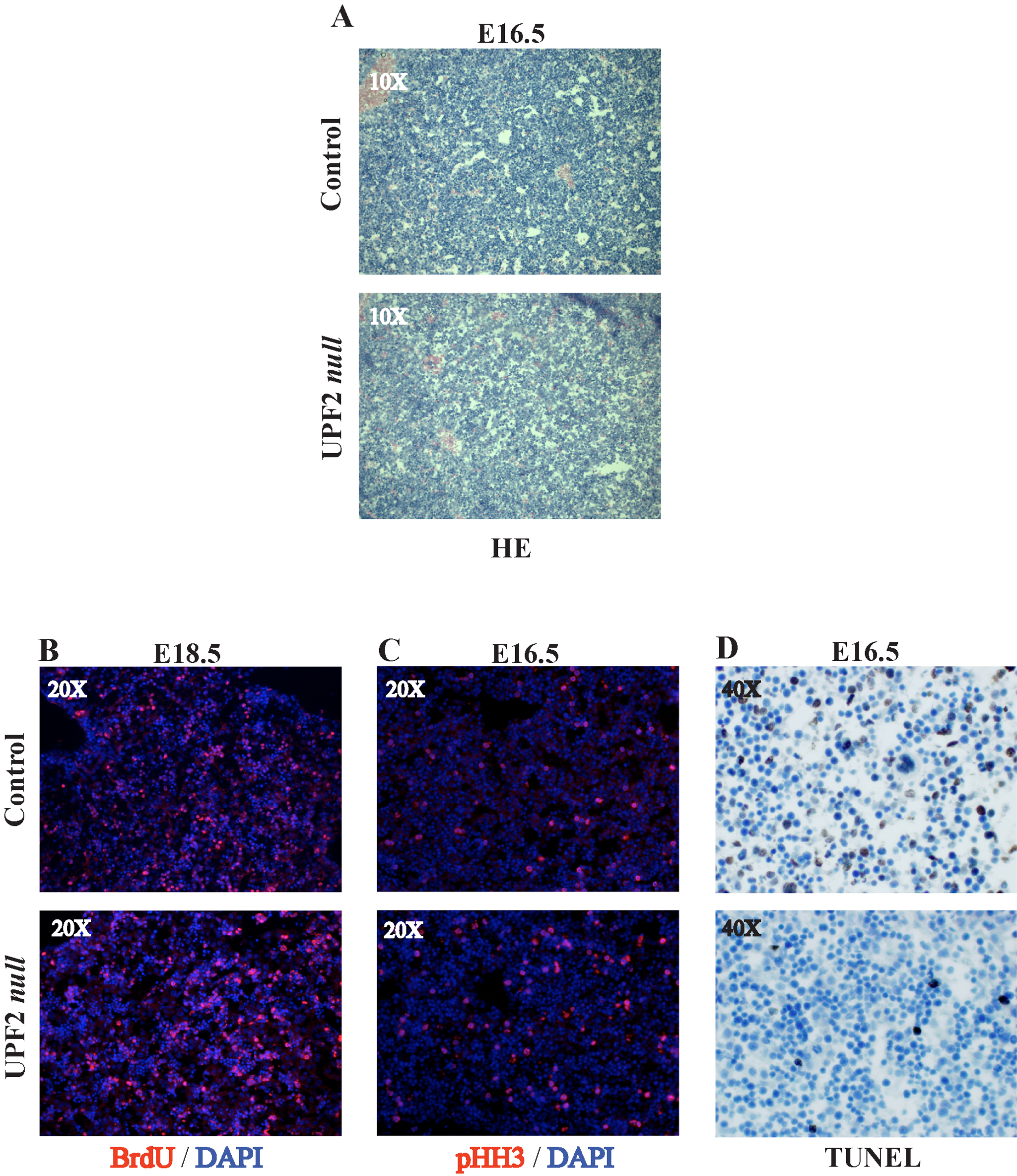

Supplement: Figure S1 — Loss of UPF2 during fetal liver development does not affect proliferation rates, number of mitotic cells or apoptosis. (A) Histological analysis of control (Upf2fl/f) and UPF2 null (Upf2fl/fl; Alfp-Cre) E16.5 fetal livers stained with HE. (B) E18.5 UPF2 null fetal livers incorporate BrdU to similar extent as control livers. (C) E16.5 UPF2 null fetal livers have similar number of mitotic cells as assayed by phosphorylation of S10 of Histone H3. (D) TUNEL staining of E16.5 UPF2 null and control fetal livers. (5.38 MB TIF) [file pone.0011650.s001.tif]

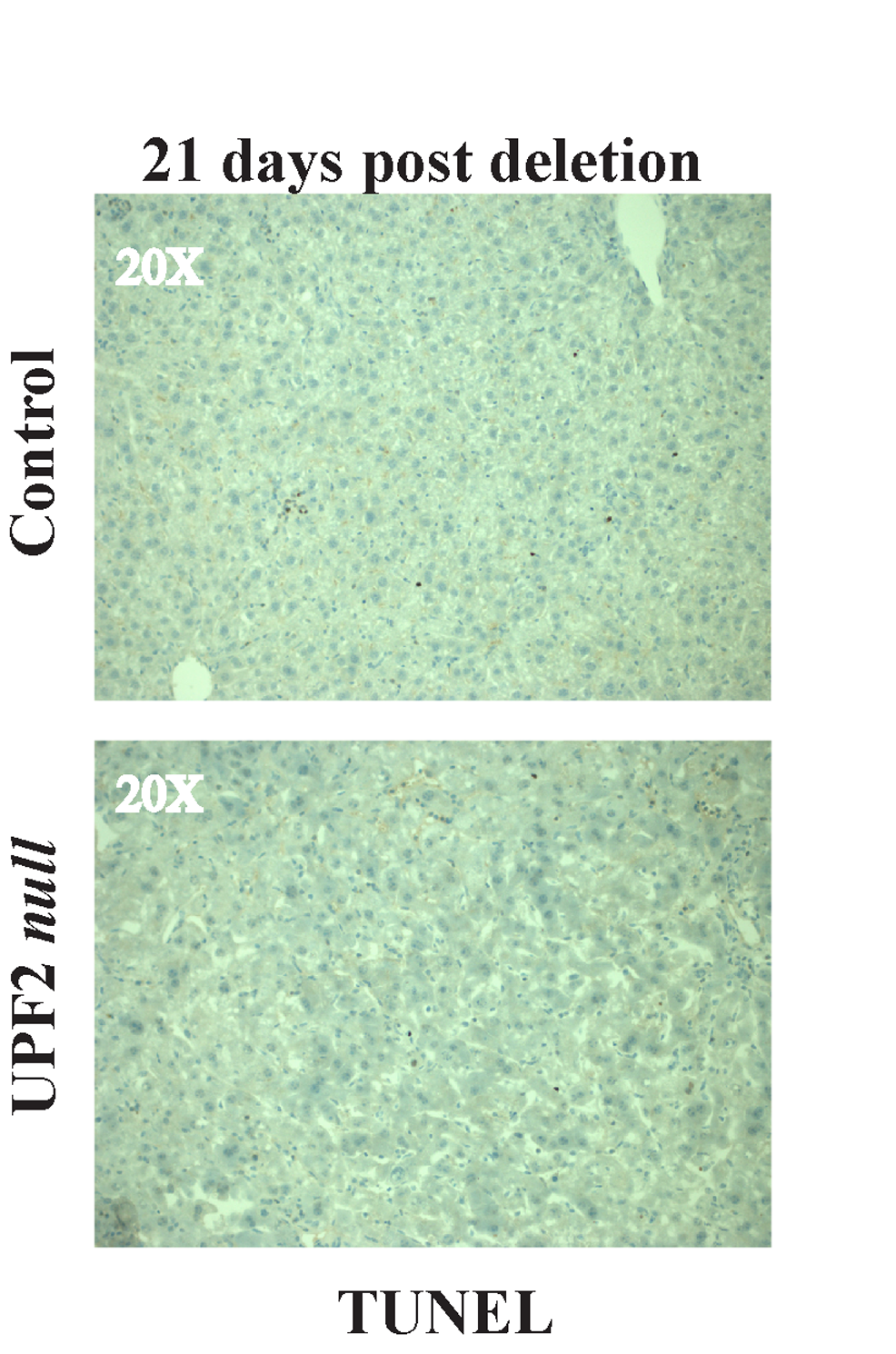

Supplement: Figure S2 — Loss of UPF2 in adult liver does not induce apoptosis. TUNEL staining of adult control and UPF2 null livers harvested 21 days post deletion. (1.77 MB TIF) [file pone.0011650.s002.tif]

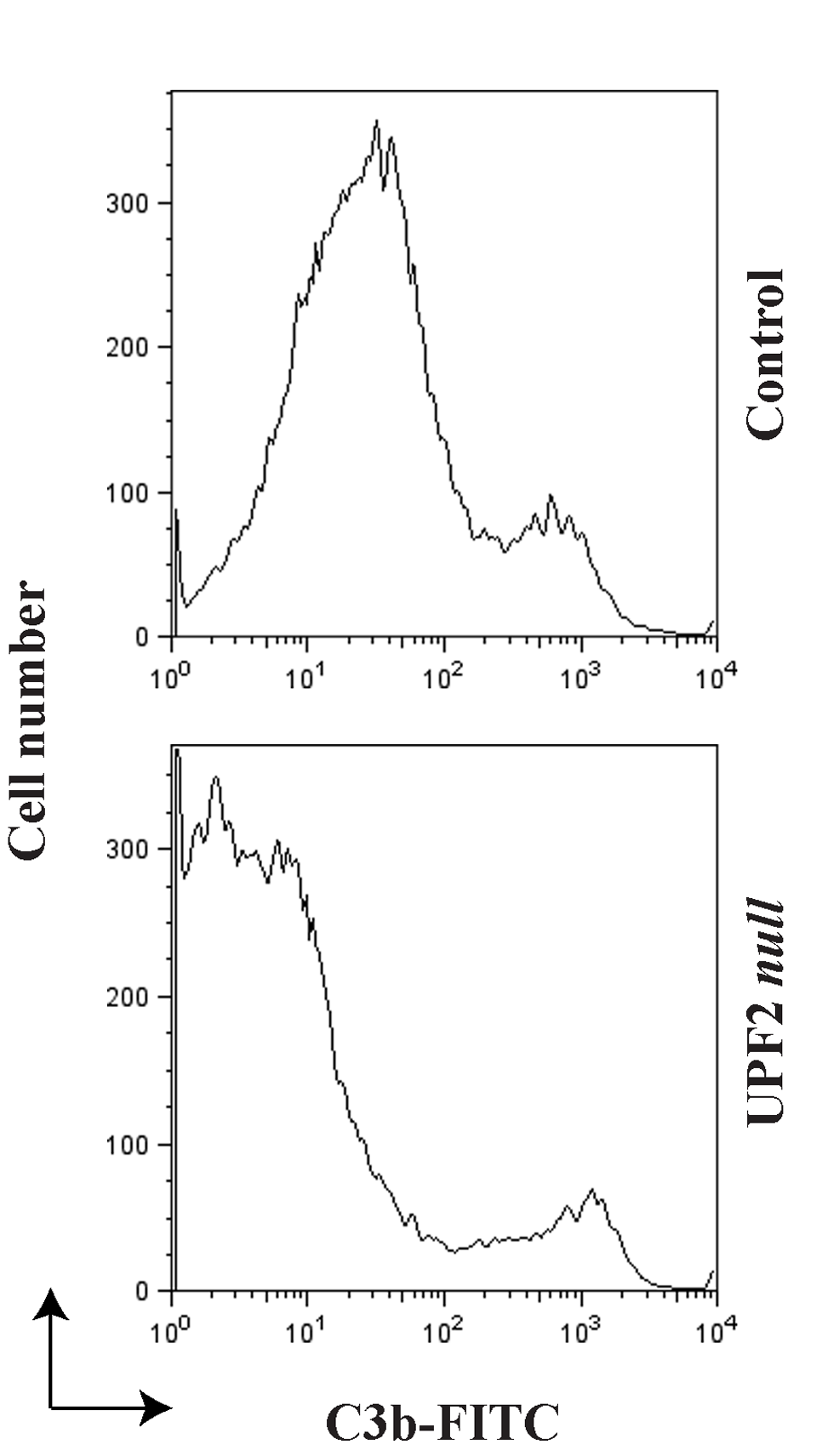

Supplement: Figure S3 — Loss of UPF2 functionally compromises the classical complement pathway. FACS analysis of C3b deposition on K562 opsonised with antibodies against K562 using sera from control (Upf2fl/fl) and UPF2 null (Upf2fl/fl;Mx1Cre) mice. Serum was harvested 2 weeks post deletion of UPF2. (0.20 MB TIF) [file pone.0011650.s003.tif]

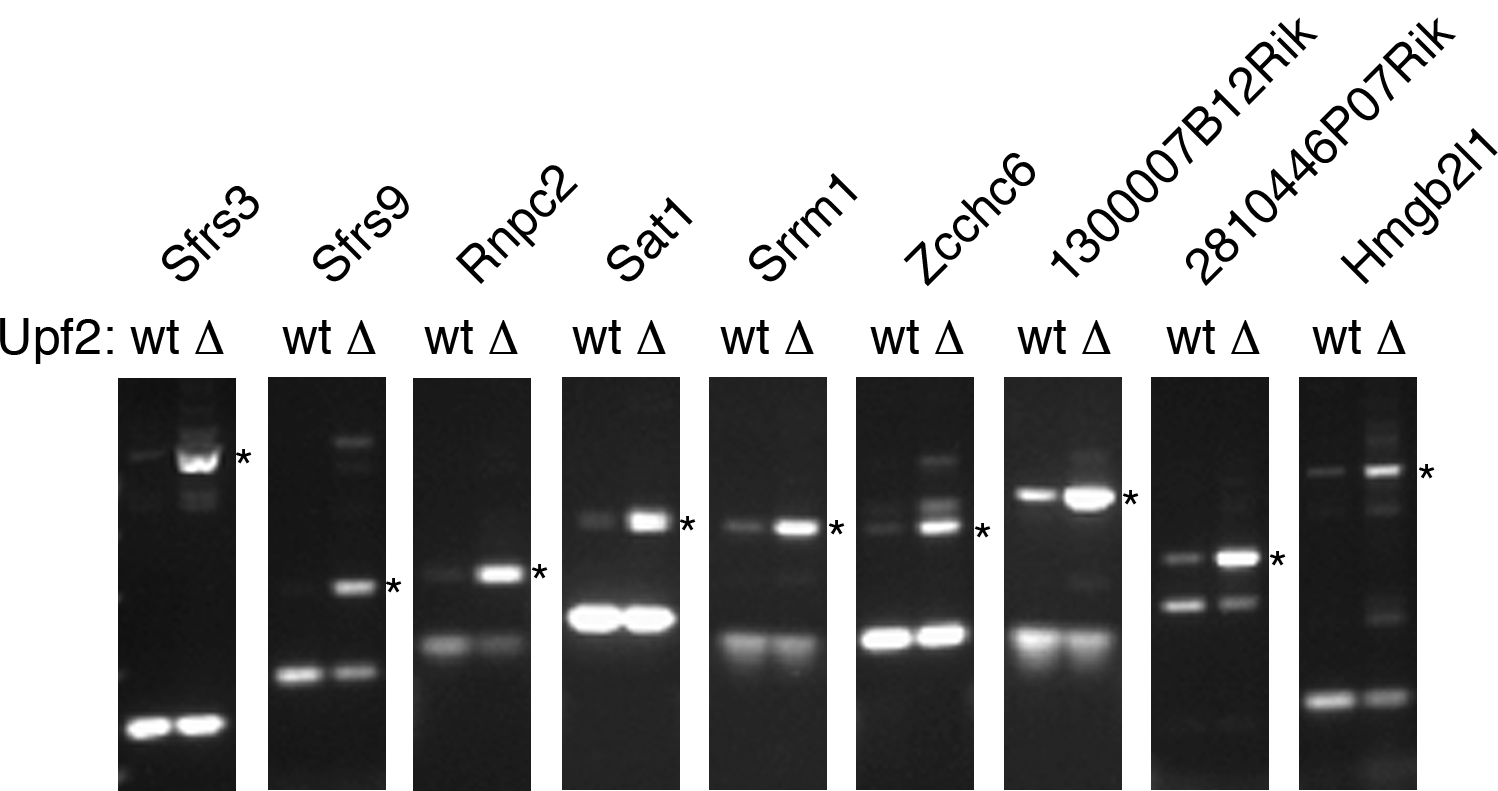

Supplement: Figure S4 — Loss of UPF2 in adult liver leads to altered splicing patterns. RT-PCR analysis of RNA isolated from adult control (Upf2fl/fl) and UPF2 null (Upf2fl/fl; Mx1Cre) livers shows that PTC-containing alternative spliced isoforms were stabilized for 9/9 tested genes. Up-regulated splice isoforms are indicated by an asterisk. (0.49 MB TIF) [file pone.0011650.s004.tif]

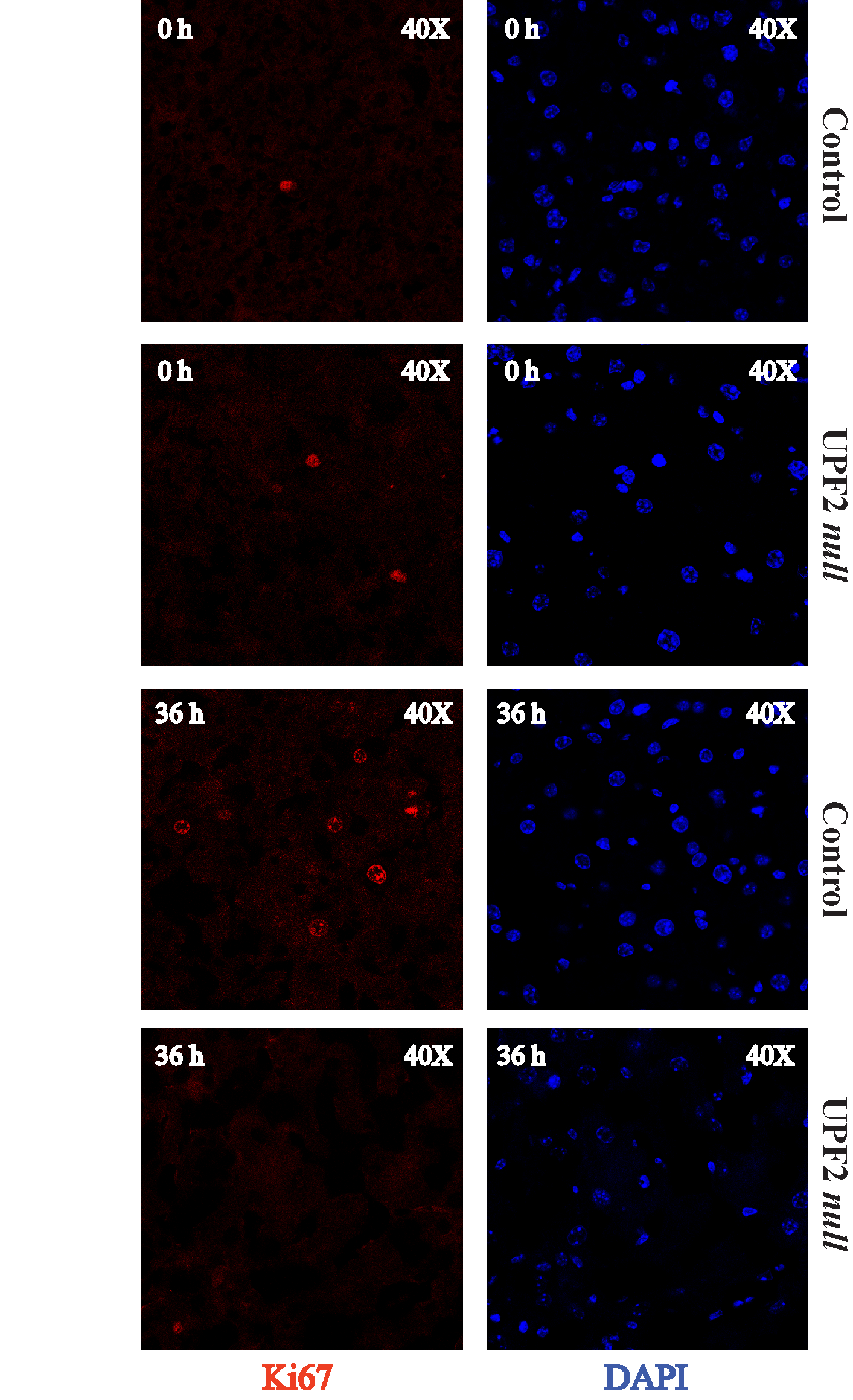

Supplement: Figure S5 — UPF2 is essential for liver regeneration. Immunofluorescence analysis of Ki67 expression before (0 h) or after (36 h) PH of control and UPF2null BM transplanted mice. (2.18 MB TIF) [file pone.0011650.s005.tif]
