# Supplementary material for: UPF2 Is a Critical Regulator of Liver Development, Function and Regeneration
Source: PLoS One. 2010 Jul 19;5(7):e11650. doi: 10.1371/journal.pone.0011650 (PMC2906512; doi:10.1371/journal.pone.0011650)
Supplement: Table S2 — SnoRNAs host genes and resident SnoRNAs. SnoRNA host genes showing up-regulation in the indicated tissues upon UPF2 truncation are depicted. (0.08 MB DOC) [file pone.0011650.s007.doc]

# Table S2: SnoRNAs host genes and resident SnoRNAs

| **Probeset** | **Gene** | **Entrez #** | **Tissue** | **SnoRNAs** |
| --- | --- | --- | --- | --- |
|  |  |  |  |  |
| 1436040_at | 2310005L22Rik | 69471 | BMM/Thy | SnoRA16, SnoRA44, SnoRA61, SnoRD99 |
| 1424977_AT | 4930418G15Rik | 69312 | Liver (adult) | SnoRD87 |
| 1437980_AT | 9130230N09Rik | 77671 | Liver (adult) | SnoRA63 |
| 1450986_AT | Nol5 | 55989 | Liver (adult) | 2 x SnoRD70 |
| 1455035_S_AT  1426533_AT | Nol5a | 67134 | Liver (adult) | SnoRD110, SnoRD51, SnoRD56, SnoRD57 |
| 1455643_S_AT | Tsr1 |  | Liver (adult) | 2 x SnoRD91 |
| 1433656_A_AT | Gnl3 | 30877 | Liver (adult) | SnoRD19, SnoRD69 |
| 1433697_AT | Patl1 | 225929 | Liver (adult) | SnoRA17 |
| 1454663_AT  1454664_A_AT  1433631_AT | Eif5 | 217869 | Liver (adult) | SnoRA28 |
| 1454915_at | Rab3gap2 | 98732 | Liver (adult) | SnoRA36 |
| 1449278_AT | Eif2ak3 /// LOC100047634 | 13666 | Liver (adult) | SnoRA5 |
| 1455600_AT | RPS3 | 27050 | Liver (adult) | 2 x SnoRD15 |
| 1433502_S_AT | Tsr1 | 104662 | Liver (adult) | 2 x SnoRD91 |
| 1451254_AT | Ikbkap | 230233 | Liver (adult) | SnoRA42 |
| 1436157_AT  1436156_AT | Ccar1 | 67500 | Liver (adult) | SnoRD98 |
| 1426626_AT | Gtf2f2 | 68705 | Liver (adult) | SnoRA45 |
| 1415985_AT | Sf3b3 | 101943 | Liver (adult) | 2 x SnoRD111 |
| 1416171_AT | 2310037I24Rik | 69612 | Liver (adult) | 2 x SnoRA2 |
| 1428390_AT | Wdr43 | 72515 | Liver (adult) | SnoRD92, 2 x SnoRD53 |
| 1418504_AT  1418503_AT | Hspa9 | 15526 | Liver (adult) | SnoRD63 |
| 1429521_AT | Alkbh8 | 67667 | Liver (adult) | SnoRA24 |
| 1435534_A_AT  1455357_X_AT | Tomm20 | 67952 | Liver (adult) | SnoRA14 |
| 1433928_A_AT  1417608_A_AT | Rpl13a | 22121 | Liver (adult) | SnoRD34, SnoRD35, 2 X SnoRD33 |
| 1455789_X_AT | Hspa8 |  | Liver (adult) | 3 x SnoRD14 |
| 1417658_at  1448796_S_AT  1448795_A_AT  1442793_S_AT | Tbrg4 | 21379 | Liver (adult)/BMM | Two copies SnoRA5 |
| 1430271_X_AT  1430147_A_AT | Josd3 | 75316 | Liver (adult/E16.5/E18.5) | SnoRA40, SnoRA18, SnoRA8, SnoRA1, SnoZ40, SnoRA32, SnoRA25 |
| 1434574_AT | 9430008C03Rik | 68108 | Liver (adult/E16.5/E18.5) | SnoRA71, SnoRA61, SnoRA71 |
| 1460672_AT | 2410002F23Rik | 66976 | Liver (adult/E16.5/E18.5) | 4 x SnoRD88 |
| 1455010_at | 1500012F01Rik | 68949 | Liver (adult/E16.5/E18.5)/  BMM/Thy | Three copies SnoRD12 |
| 1449410_a_at  1419291_x_at | Gas5 | 14455 | Liver (adult/E16.5/E18.5)/  BMM/Thy | SnoZ18, SnoRD24, SnoRD79, snoRNA snR60, SnoRD47, SnorRD81 |
| 1436506_a_at | Snhg6 | 73824 | Liver (adult/E16.5/E18.5)/  BMM/Thy | SnoRD87 |
| 1435524_at | 2010109N14Rik | 69895 | Liver (adult/E16.5/E18.5)/  BMM/Thy | SnoRA24 |
| 1419553_a_at | Rabggtb | 19352 | Liver (adult/E16.5/E18.5)/  BMM | Three copies SnoRD45 |
| 1428529_at | 2810026P18Rik | 72655 | Liver (adult/E16.5/E18.5)/ BMM/Thy | Two copies SnoRD49 |
| 1453344_at | 1810032O08Rik | 66293 | Liver (adult/E16.5/E18.5)/ BMM/Thy | SnoRD38 |
| 1439399_a_at  1433675_at  1433674_a_at | Snord22 | 83673 | Liver (adult/E16.5/E18.5)/ BMM/Thy | SnoRD25, SnoRD26, SnoRD27, SnoRD28, SnoRD29, SnoRD30, SnoRD31, SnoRD22 |
| 1447936_at | 2410006H16Rik | 69221 | Liver (adult/E16.5/E18.5)/ BMM/Thy | Two copies SnoRD49 |
| 1434660_AT | Alkbh1 | 211064 | Liver (adult/E18.5) | SnoRA58 |
| 1450934_at | Eif4a2 | 13682 | Liver (E16.5/E18.5) | SnoRA4, 2 x SnoRA63 |
| 1426880_at | Etl4 | 208618 | Liver (E18.5) | SnoRA32 |
| 1441081_a_at | 1110038B12Rik | 68763 | Thy | SnoRD48, SnoRD52 |
| 1437538_at | 2610002F03Rik | 72091 | Thy | SnoRA43 |

SnoRNA host genes showing up-regulation in the indicated tissues upon UPF2 truncation are depicted. The following criteria were used:

Adult liver: > 1.5 fold (*P*<0.001)

E16.5/E18.5 liver: > 2.0 fold (*P*<0.05)

Thymus (Thy): > 2.0 fold (*P*<0.05)

Bone Marrow derived macrophages (BMM): > 2.0 fold (*P*<0.05)

Data from Thy and BMM are from [13] (see primary manuscript)
