# Supplementary material for: UPF2 Is a Critical Regulator of Liver Development, Function and Regeneration
Source: PLoS One. 2010 Jul 19;5(7):e11650. doi: 10.1371/journal.pone.0011650 (PMC2906512; doi:10.1371/journal.pone.0011650)
Supplement: Table S3 — Average affymetrix gene expression fold changes for selected genes. In case of multiple probesets the set with the highest expression levels are shown. (0.04 MB DOC) [file pone.0011650.s008.doc]

**Supplemental Table S3. Average affymetrix gene expression fold changes for selected genes.**

| **Probeset** | **Gene** | **Fold change**  **UPF2 *null vs* control** |
| --- | --- | --- |
| 1427001_s_at | Hnf4a | -1.49 |
| 1448261_at | Cdh1 | 1.00 |
| 1460681_at | Ceacam1 | -1.29 |
| 1448767_s_at | Gjb1 | -1.96 |
| 1423271_at | Gjb2 | 1.04 |
|  | | |
| None of these changes were statistical significant | | |

| **PH in control and UPF2 *null* cells** | | | |
| --- | --- | --- | --- |
| **Probeset** | **Gene** | **Fold change**  **36h vs 0h**  **Control** | **Fold change**  **36h vs 0h**  **UPF2 *null*** |
| 1455899_x_at | Socs3 | 5.22 (*) | 4.83 (*) |
| 1449326_x_at | Saa2 | 30.68 (*) | 6.19 (*) |
| 1418982_at | Cebpa | -1.93 (*) | -2.27 (*) |
| 1427844_a_at | Cebpb | 2.07 | 1.31 |
|  | | | |
| (*) p<0.05 | | | |

In case of multiple probesets the set with the highest expression levels are shown.
