# Supplementary material for: UPF2 Is a Critical Regulator of Liver Development, Function and Regeneration
Source: PLoS One. 2010 Jul 19;5(7):e11650. doi: 10.1371/journal.pone.0011650 (PMC2906512; doi:10.1371/journal.pone.0011650)
Supplement: Table S9 — Primers used in this study. (0.07 MB DOC) [file pone.0011650.s014.doc]

**Supplemental Table S9:** Primers used in this study.

| **qRT-PCR primer list** | | **RT-PCR primer list for AS targets** | |
| --- | --- | --- | --- |
| **Gene** | **Sequence 5’-3’** | **Gene** | **Sequence 5’-3’** |
| Sfrs2_For | CGAAGATCCAAGTCCAAGTC | Sfrs3_For | TGATTACCGCAGGAGGAGTC |
| Sfrs2_Rev | CCAATGTCCTCTGTTAAGCC | Sfrs3_Rev | GATCGAGACGGCTTGTGATT |
| Sfrs16_For | CGCTACAGTCGAGAGTACAG | Sfrs9_For | GCCTCCTACAAGACGGTCAG |
| Sfrs16_Rev | TCACTACAGAAACCCAGCAG | Sfrs9_Rev | TATTCAACCATCCCCATTCC |
| Sfrs12_For | ACACAAGAAACGCTCTAAGTC | Rnpc2_For | TATTGAAGCCATGCTTGAGG |
| Sfrs12_Rev | CTTCACTCTTTCCTTCACCT | Rnpc2_Rev | TCTTCATGGCCGTTAGCACT |
| Ptbp2_For | TATACTCGACCTGATCTGCC | Sat1_For | GAGGATGGCTTTGGAGAACA |
| Ptbp2_Rev | GGAATAGCCAAAGGACTGAG | Sat1_Rev | CCAATCCATGGGTCATAGGT |
| Adhfe1_For | TTGACCTAAATGTTGATGACGG | Srrm1_For | TCAACCAGCTGGAAGTGAAG |
| Adhfe1_Rev | GTGAGTTAAGTGTGGAGATAAGAC | Srrm1_Rev | ACCCAGTCAGGTTGATTTGC |
| Fasn_For | ACAACCTCTTCCTGTTTGAC | Zcchc6_For | ACCAGCTCAGCAAAAGAGGA |
| Fasn_Rev | TTAGTGATAAGGTCCACGGAG | Zcchc6_Rev | CAGCAATTCCACCCAGAGTT |
| Acsl5_For | TTTATTCCAGGAGCAGACCA | 1300007B12Rik_For | CACTCCCAGCACACTGAAGA |
| Acsl5_Rev | CACAGTTCTTCAAATGATCCC | 1300007B12Rik_Rev | CCAACAATCTTTCTGCACCA |
| Mcm2_For | TCAGAGAATGTAGACCTCAC | 2810446P07Rik_For | CAGTGCAATGCAGTTTCTTGA |
| Mcm2_Rev | GATATACTTCTTCAGCACCTCC | 2810446P07Rik_Rev | TTCACCCAGAGCAGAACCAT |
| Ccne2_For | GGAAGATAGACACAATATCCAGAC | Hmgb2l1_For | AAGCACTCCCCTGACGATTA |
| Ccne2_Rev | AGAATGGCAGTGTAACTCCT | Hmgb2l1_Rev | TTTCAACAGGTCCATTGCTG |
| C1s_For | GTAAAGTTGAAGAGCCAGAG | Sfrs3_For | TGATTACCGCAGGAGGAGTC |
| C1s_Rev | GGTTGTCCTCCAAATATCCT | Sfrs3_Rev | GATCGAGACGGCTTGTGATT |
| Rik181_For | CTCTATCTGGTTATTGCCCTG | Sfrs9_For | GCCTCCTACAAGACGGTCAG |
| Rik181_Rev | AGAACAAACTCCAAGGTCGT | Sfrs9_Rev | TATTCAACCATCCCCATTCC |
| Sngh1_For | GCCAGGCCTGTTCAATTTTA |  |  |
| Snhg1_Rev | TGCCTGAGATTTGTCACCAG | **qPCR primers for ChIP** | |
| C2_For | GGACCTGAGTGGACAAGGTG | Nol5_For | TCAGAAATCTGCCCACCTCTGT |
| C2_Rev | CCCACCTGGAAGAACCTGTA | Nol5_Rev | TGCTTAATTTAGGGGCTGCTCAA |
| C8b_For | TTAAGATCAAGGCTGAGCCC | 9130230N09Rik_For | AATGCCTTTGACGGGTCTCTTG |
| C8b_Rev | GATCCTTTCAGGACAGGGAC | 9130230N09Rik_Rev | GCCTGGCCCTTCATGGATG |
| Hmgsc1_For | CAATAGACTCACTCTTTGAAGG | 1810032O08Rik_For | TGTGGTAATGGCTGCCCTTCA |
| Hmgcs1_Rev | TTGGAATATGCTCTGTTGCT | 1810032O08Rik_Rev | GGTACAGGTCACCCACTACA |
| Acat2_For | CCTTCTGTTATGGGAGTAGGACCA | Rabggtb_For | CATTTGGGCCTATGATTCCCACT |
| Acat2_Rev | CATCGATGTTGACCTTCTCG | Rabggtb_Rev | TGTCTTAGAGAGCGTGGCATTTG |
| Ccnd1_For | CTCCCCACGATTTCATCGAACAC | 1500012F01Rik_For | GGGCAGTGATAGGGAATTTGATG |
| Ccnd1_Rev | AAATGAACTTCACATCTGTGGC | 1500012F01Rik_Rev | ACGAACCTCAGCAGTAACTCTTG |
| Ccna2_For | ACCCGTACCTTAAGTACCTG | Gas5_For | GACGATTGCTCTTGGGAAGTAC |
| Ccna2_Rev | TAAGACTCTCCAGGGTATATCCA | Gas5_Rev | GGCACTGTTGAGTTGCACATATC |
| rpPO_For | TTCATTGTGGGAGCAGAC | Actb_For | gatagttcgccatggatgacgat |
| rpPO_Rev | CAGCAGTTTCTCCAGAGC | Actb_Rev | GACGACCAGCGCAGCGATA |
|  |  | Chr12-des_For | TAGCTTTCGACAGAGGTCCTAAG |
| **PCR primers for sex determination** | | Chr12-des_Rev | CCGAAGGTGGCCGGTTGT |
| SRYs | GCTTGACAGTATCTAGGTTCA |  |  |
| Sryas | GACCACACCATAAATGCATTCA |  |  |
